# Joint mobilization combined with cervical resistance training for pediatric atlantoaxial rotatory subluxation: Protocol for a randomized controlled trial with musculoskeletal ultrasound assessment

**DOI:** 10.1371/journal.pone.0359198

**Published:** 2026-09-24

**Authors:** Ge Cai, Yi Sun, Shuaizi Yin, Shuaiyu Ying, Huasong Luo, Yuanyuan Li, Ting Wu, Haoyang Zhang

**Affiliations:** 1 Department of Tuina, Hangzhou Hospital of Traditional Chinese Medicine Affiliated to Zhejiang Chinese Medical University, Hangzhou, Zhejiang, China; 2 Department of Tuina, Hangzhou School of Clinical Medicine, Zhejiang Chinese Medical University, Hangzhou, Zhejiang, China; Hamad Medical Corporation, QATAR

## Abstract

**Background:**

Atlantoaxial rotatory subluxation (AARS) is a common cause of acquired painful torticollis in children. Conservative treatment is widely used, but high-quality randomized evidence for structured muscle-focused rehabilitation is limited. Musculoskeletal ultrasound, especially shear-wave elastography (SWE), can measure cervical muscle stiffness in a non-invasive way. This trial aims to test the efficacy and safety of joint mobilization combined with cervical resistance training in children with AARS. It will also examine whether ultrasound-based measures can detect treatment-related changes in cervical muscle stiffness.

**Methods:**

This is a single-center, assessor- and analyst-blinded, randomized controlled superiority trial with two parallel groups. Seventy children aged 2–18 years with clinically and radiologically confirmed AARS will be randomly assigned in a 1:1 ratio to joint mobilization plus cervical resistance training or joint mobilization alone. Both groups will receive treatment 3 times per week for 4 weeks. The primary outcome is affected-side sternocleidomastoid stiffness at week 4 measured by SWE and adjusted for baseline stiffness. Secondary outcomes include bilateral sternocleidomastoid and upper trapezius SWE parameters, pain intensity, cervical disability, cervical rotation range of motion, overall clinical efficacy, and recurrence-related outcomes. SWE outcomes will be assessed at baseline, week 2, and week 4. Pain and disability outcomes will be assessed at baseline, week 2, week 4, and at 3 and 6 months after treatment. Cervical rotation range of motion will be assessed at baseline, week 2, and week 4. Recurrence-related outcomes will be recorded at the 3- and 6-month follow-up visits.

**Discussion:**

This randomized controlled trial is designed to provide high-quality evidence regarding the effectiveness of joint mobilization combined with cervical resistance training for pediatric AARS. By incorporating musculoskeletal ultrasound as an objective assessment tool, the study aims to elucidate the relationship between cervical muscle mechanical properties and clinical outcomes. The findings may contribute to the development of standardized, muscle-focused conservative treatment strategies for children with AARS.

**Trial registration:**

International Traditional Medicine Clinical Trial Registry (ITMCTR2025002448), registered on 27 October 2025.

## Introduction

Atlantoaxial rotatory subluxation (AARS) is a cervical disorder in which the atlas (C1) rotates abnormally or becomes fixed relative to the axis (C2). The condition often causes painful torticollis, abnormal head posture and restricted neck movement [1–3]. A characteristic physical sign is the so-called “cock-robin” posture, in which the head is tilted to one side with the chin rotated towards the opposite side [4]. Epidemiological studies indicate that atlantoaxial disorders are proportionally more common in pediatric populations than in adults, with AARS representing one of the most frequent cervical spine conditions in children, particularly those under 12 years of age [5]. It may develop after infection, inflammation, trauma, or otolaryngological procedures. Grisel’s syndrome refers to a non-traumatic form of AARS that occurs secondary to upper airway infection, inflammation, or recent head and neck surgery, especially otolaryngological procedures [2,6]. Delayed recognition may increase treatment difficulty and may lead to more invasive management [3,7,8].

The atlantoaxial joint is the most mobile part of the cervical spine and contributes about 60% of total cervical rotation [9]. Children are more prone to AARS because of developmental features such as greater ligamentous laxity, immature bony anatomy, shallow articular surfaces, and relatively limited upper cervical stability [10]. In many children, mild trauma, upper respiratory infection, or local inflammation can disturb atlantoaxial mechanics and lead to rotational fixation rather than a complete dislocation [3,11]. Abnormal atlantoaxial alignment may disturb cervical biomechanics and induce pain, muscle guarding, and compensatory posturing. As cervical stability depends on the integrated function of joints, ligaments, and muscles, joint dysfunction may also impair deep cervical muscle control and promote compensatory overactivity of superficial muscles such as the sternocleidomastoid and trapezius [7,12]. This mechanical change may increase pain, muscle guarding, and local soft-tissue stress. It may also disturb normal movement control around the neck.

Conservative treatment is the main approach for most non-surgical cases of pediatric AARS [1,3,8]. Common options include rest, analgesia, cervical collar immobilization, traction, and manual therapy. Many children improve with these measures, but treatment response is not always stable, and some children continue to show pain, limited motion, or recurrent symptoms [11–14]. Because of this, clinicians still need rehabilitation programs that can better restore muscle control and cervical stability after joint alignment improves.

Joint mobilization is often used in conservative care for cervical dysfunction and neck pain [15–17]. This method may reduce pain, improve segmental mobility, ease protective muscle guarding, and help restore more normal movement. Cervical resistance training may improve muscle activation, endurance, and motor control. These effects may support recovery after joint malalignment is corrected or reduced. However, most evidence for mobilization and exercise comes from other cervical disorders or adult populations rather than pediatric AARS [15,18,19]. This supports the need for a pediatric randomized trial.

Musculoskeletal ultrasound is being used more often in pediatric rehabilitation because it is safe, non-invasive, radiation-free, and suitable for repeated bedside assessment [20,21]. In cervical disorders, ultrasound can show muscle size, symmetry, and soft-tissue status. Shear-wave elastography can also quantify muscle stiffness and provide a more objective view of tissue change. These features make ultrasound a useful tool for tracking recovery and studying treatment effects in AARS [22–24].

Randomized evidence specific to pediatric AARS remains limited, and objective markers of muscle recovery are rarely incorporated into clinical studies in this field. This study was designed to address both gaps by combining a randomized comparison of two conservative treatment strategies with ultrasound-based outcome assessment [23,25,26].

The primary objective is to determine whether joint mobilization combined with cervical resistance training is superior to joint mobilization alone for reducing affected-side sternocleidomastoid muscle stiffness at week 4. Secondary objectives are to compare the effects of the two interventions on pain, cervical disability, cervical rotation range of motion, additional SWE parameters in the sternocleidomastoid and upper trapezius muscles, recurrence-related outcomes during follow-up, and the occurrence of intervention-related adverse events.

## Methods

### Study design

**Fig 1:**
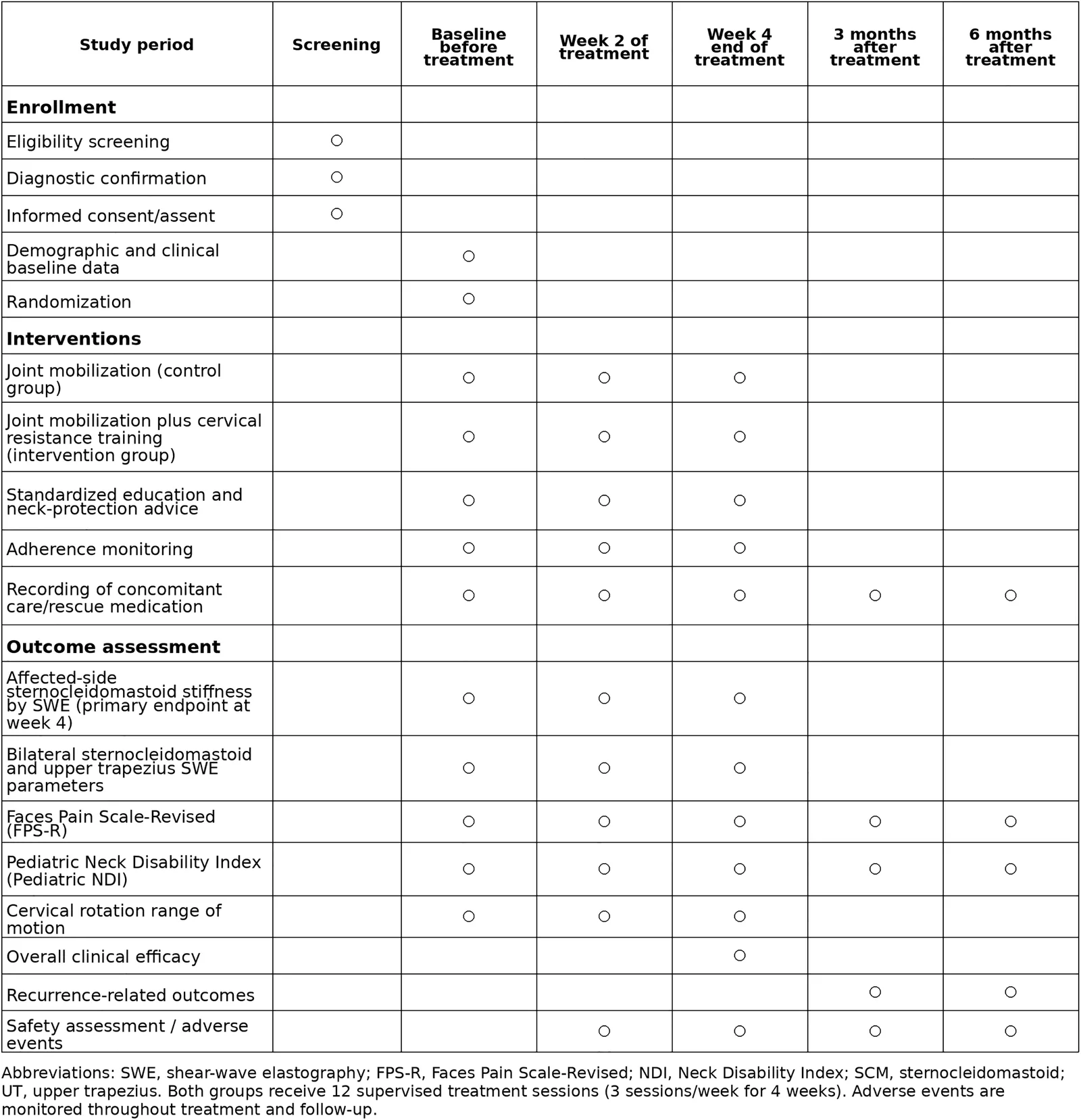
Schedule of enrollment, interventions, and assessments. The figure presents the planned time points for enrollment, allocation, treatment sessions, outcome assessments, and follow-up visits.

**Fig 2:**
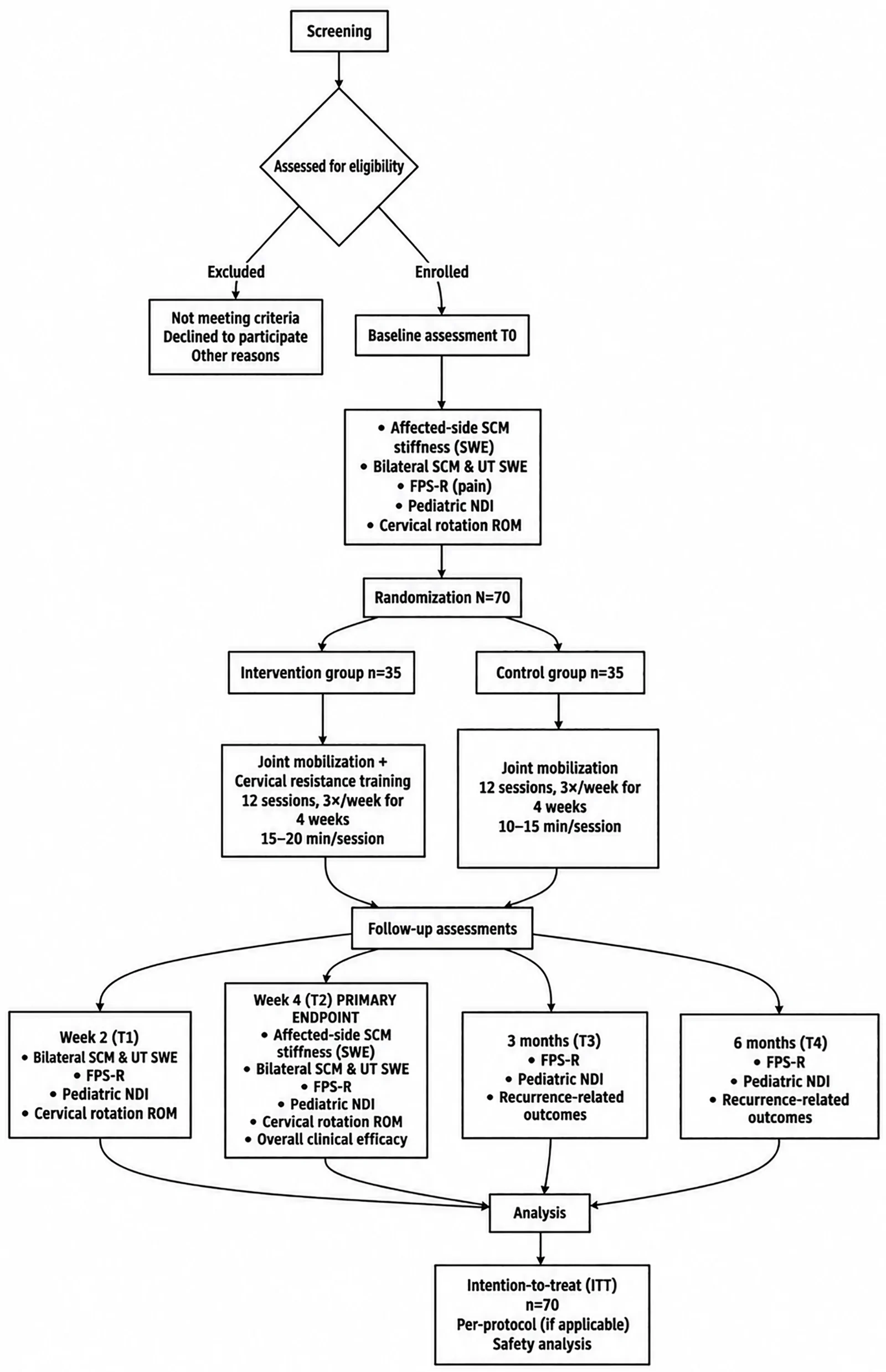
Study flow diagram. The figure summarizes screening, randomization, intervention delivery, outcome assessment, follow-up, and analysis procedures for the randomized controlled trial.

This study is a single-center, randomized, controlled, two-arm, parallel-group superiority trial. Outcome assessors, ultrasound evaluators, and statisticians will remain blinded to group allocation. Because the two interventions differ clearly in content and delivery, participants and treating therapists cannot be blinded. Fig 1 shows the schedule of enrollment, interventions, and assessments, and Fig 2 shows the study flow.

### Study setting

The trial will take place in the Department of Tuina, Hangzhou Hospital of Traditional Chinese Medicine in Hangzhou, China. Potential participants will be recruited consecutively through outpatient screening, inpatient consultation, and referral from related departments at the study site until the target sample size is reached. Investigators will regularly review screening logs and recruitment progress to support timely enrollment.

### Participants

#### Diagnostic criteria.

Eligible participants must meet both clinical and imaging criteria for AARS. Clinical diagnosis will be based on painful torticollis, abnormal head posture, and limited cervical rotation. Imaging confirmation will be based on cervical radiography and/or computed tomography demonstrating atlantoaxial rotational asymmetry or rotatory subluxation, as interpreted by the treating clinical team according to routine institutional practice. When necessary, additional imaging may be used to support diagnosis and exclude alternative cervical pathology [27,28].

#### Inclusion criteria.

(1) age 2–18 years;
(2) clinically and radiologically confirmed AARS;
(3) stable general condition, ability to follow simple instructions, and ability to complete the intervention and outcome assessments;
(4) written informed consent from a legal guardian.

#### Exclusion criteria.

(1) congenital cervical malformation, cervical spine fracture, spinal tumor, spinal infection, inflammatory cervical instability that needs disease-specific treatment, or severe neurological disease;
(2) previous cervical spine surgery;
(3) severe traumatic AARS, progressive neurological deficit, or another condition that requires urgent reduction or surgical treatment;
(4) severe cardiopulmonary disease or another systemic disorder that prevents participation;
(5) inability to cooperate with ultrasound examination or functional assessment;
(6) other structured cervical rehabilitation, manual therapy, traction, acupuncture, or invasive cervical treatment within 2 weeks before enrollment; or
(7) participation in another clinical trial within the previous 3 months.

#### Withdrawal criteria.

Participants may be withdrawn if a legal guardian withdraws consent, if the child cannot continue the intervention or key outcome assessments, if a serious adverse event or clear clinical deterioration requires another treatment, or if a major protocol violation makes continued participation inappropriate. The reason and timing of withdrawal will be recorded. When possible, data collected before withdrawal will be kept for intention-to-treat analyses in line with consent and local regulations.

### Ethical approval and informed consent

This study was approved by the Ethics Committee of Hangzhou Hospital of Traditional Chinese Medicine (Approval No. 2024KLL088). The trial will be conducted in accordance with local legislation, institutional requirements, and the principles of the Declaration of Helsinki. Written informed consent will be obtained from legal guardians by a trained investigator before any study-specific procedures. Assent will be sought from children when appropriate using age-appropriate explanations. Important protocol modifications will be submitted to the ethics committee for approval before implementation unless an immediate change is required for participant safety.

Relevant protocol amendments will also be updated in the trial registry and communicated to investigators, participants, and legal guardians as appropriate. Participants who experience harm or clinical deterioration during the trial will receive appropriate medical evaluation and treatment according to usual clinical practice. No special ancillary care or financial compensation beyond standard institutional and legal arrangements is planned. Study findings will be disseminated through peer-reviewed publications, academic conferences, and trial registry updates where appropriate. A plain language summary will be available to participating children and their legal guardians on request. No biological specimens will be collected, and no ancillary studies requiring additional consent are planned.

### Randomization and allocation concealment

Participants will be randomly assigned in a 1:1 ratio to the intervention group or the control group. The random allocation sequence will be generated by an independent statistician using computer-generated block randomization. Group assignments will be placed in sequentially numbered, opaque, sealed envelopes prepared by personnel not involved in recruitment, treatment or outcome assessment. After baseline assessment and confirmation of eligibility, the next envelope in sequence will be opened to reveal treatment allocation.

### Implementation

Potential participants will be screened and enrolled by study investigators at the trial site who are responsible for recruitment and baseline assessment. These investigators will not have access to the random allocation sequence in advance. The random sequence will be generated by an independent statistician and implemented through sequentially numbered, opaque, sealed envelopes. After eligibility confirmation and completion of baseline assessment, the next envelope in sequence will be opened by designated study personnel to assign the participant to the allocated intervention. Treating therapists and outcome assessors will not have prior access to the allocation sequence.

### Blinding

Because the two interventions differ clearly in content and delivery, blinding of participants and treating therapists is not feasible. To minimize bias, outcome assessors, ultrasound examiners, and statisticians will remain unaware of treatment allocation, and participants and their guardians will be reminded not to disclose group assignment during outcome assessments.

Unblinding of blinded study personnel will be permitted only when knowledge of treatment allocation is necessary for participant safety or urgent clinical management. Any unblinding will be decided by the principal investigator, documented with the reason, and restricted to the minimum personnel necessary. Data analysts will remain blinded until the main analysis is completed whenever possible.

### Interventions

All interventions will be delivered by licensed therapists with formal clinical training in pediatric cervical rehabilitation and manual therapy. Before treating trial participants, all treating therapists will receive protocol-specific training on participant positioning, intervention procedures, progression rules, safety precautions, and documentation requirements. Only therapists who have completed the study training and demonstrated familiarity with the treatment manual will be permitted to deliver the study interventions. The study team will use a standardized treatment manual to improve consistency across sessions.

#### Control group: Joint mobilization.

Participants in the control group will receive joint mobilization therapy targeting the atlantoaxial joint and adjacent upper cervical segments. The procedure will include gentle traction mobilization, static segmental mobilization, and dynamic upper cervical mobilization.

During traction mobilization, participants will be placed in the supine position. The therapist will support the occipital region and mandible and will apply gentle cervical traction combined with controlled flexion, extension, lateral flexion, and rotation. Each movement will be repeated five times, with each stretch lasting approximately 6 s.

During static mobilization, participants will be placed in the sitting position. The therapist will apply rhythmic posterior-to-anterior pressure to the cervical spinous processes and bilateral transverse processes, together with vertical and lateral mobilization of the cervical segments. Each maneuver will be repeated five times, with each stretch lasting approximately 6 s.

During dynamic mobilization, participants will remain seated with the neck slightly flexed. The therapist will stabilize the occiput and apply gentle anterior gliding mobilization to C2, followed by small-amplitude rhythmic mobilization of the upper cervical segments. The end position will be maintained for at least 10 s when tolerated. Mobilization intensity will be adjusted according to each participant’s symptoms and tolerance.

Each session will last approximately 10–15 min. Treatment will be performed three times per week for 4 consecutive weeks, for a total of 12 sessions.

#### Intervention group: Joint mobilization plus cervical resistance training.

Participants in the intervention group will receive the same joint mobilization procedure as the control group, followed by structured cervical resistance training designed to improve muscle activation, neuromuscular control, and dynamic cervical stability.

Before resistance training, selective activation of the deep cervical flexor muscles will be performed in a supine crook-lying position with gentle cranio-cervical flexion while neutral cervical alignment is maintained. Light tactile cueing may be used to reduce substitution by superficial cervical muscles. This preparatory part will usually include 2–3 sets of 5 repetitions.

Manual resistance exercise will then be performed in four directions: flexion, extension, rotation, and lateral flexion [15,16,18]. All exercises will be isometric, with the cervical spine kept in a neutral position. Each contraction will be held for 5–8 s, followed by 5–10 s of rest. Participants will complete 8–10 repetitions per set and 2–3 sets in each direction. Resistance will be applied manually by the therapist. It will be progressed according to tolerance, symptom response, and movement control by adjusting therapist-applied resistance, contraction duration, or repetition number. Maximal effort testing will not be used. Training will be stopped or modified if pain rises clearly, compensatory movement becomes obvious, or the child cannot maintain the required position. Each treatment session will last about 15–20 min.

#### Frequency and duration.

Both groups will receive treatment 3 times per week for 4 consecutive weeks, for a total of 12 sessions.

#### Concomitant care and prohibited treatments.

During the intervention period, all participants will receive standardized education on posture, neck protection, and activity modification. Guardians will be advised to supervise daily behavior and to avoid excessive cervical rotation, sudden neck movements, prolonged head tilt, and other activities that may increase cervical stress or worsen symptoms. Routine daily care will be allowed as long as it does not interfere with the assigned intervention. No additional cervical immobilization will be prescribed as part of the study intervention. In the intervention group, no extra home-based neck strengthening exercises outside the study program will be allowed.

To reduce confounding, both groups will not receive other non-study cervical treatments during the intervention period. These treatments include manual therapy, traction, acupuncture, massage, structured rehabilitation outside the protocol, and invasive cervical procedures. Usual medication that is judged necessary for safety may still be used and will be recorded.

#### Adherence monitoring.

Treatment attendance will be recorded for all supervised sessions. Guardians in both groups will complete brief logs documenting cooperation with study instructions and any non-study cervical treatments. Adherence will be reviewed weekly by study staff. Important deviations from the assigned intervention, including missed treatment sessions or receipt of prohibited co-interventions, will be recorded. If major non-adherence occurs, adherence data will be summarized descriptively and considered in sensitivity analyses.

#### Protocol clarification.

The original ethics-approved clinical trial protocol in Chinese is provided as S1 File, and its complete English translation is provided as S2 File. It specified a single-center randomized controlled design, 70 participants, joint mobilization as the control intervention, joint mobilization combined with cervical resistance training as the intervention, a 4-week treatment period with 3 sessions per week, and follow-up at 3 and 6 months after treatment. In this manuscript, several reporting clarifications were made to meet clinical trial reporting standards. Affected-side sternocleidomastoid stiffness at week 4 was specified as the primary ultrasound-based endpoint based on the SWE-based cervical muscle elasticity outcome listed in the original protocol. The ultrasound analysis in this manuscript focuses on the sternocleidomastoid and upper trapezius muscles because these muscles are more directly related to torticollis presentation and can be measured more reproducibly in children. FPS-R and Pediatric NDI were used as pediatric-adapted measures of pain and neck disability, and week 2 was added as a non-primary interim assessment point to describe short-term changes during treatment. This additional assessment will not be used for the primary efficacy conclusion. These clarifications did not change the intervention framework, sample size, or overall study objective.

### Outcome measures

Outcome assessments will be conducted at baseline, week 2, week 4, and at 3 and 6 months after treatment. Week 2 will be used as an interim assessment point, while week 4 will be the end-of-treatment assessment point. The 3- and 6-month follow-up visits will be used to assess longer-term clinical outcomes and recurrence-related outcomes.

The primary outcome is affected-side sternocleidomastoid muscle stiffness at week 4 measured by shear-wave elastography and expressed as Young’s modulus (kPa). The primary comparison will be the between-group difference at week 4 after adjustment for baseline stiffness. The affected side will be defined before randomization according to the clinically dominant side of torticollis and muscle tightness, supported by baseline physical examination and imaging findings.

#### Ultrasound assessment procedures.

Ultrasound examinations will be performed using a Supersonic Aixplorer system (Supersonic Imagine, France) equipped with a 4–15 MHz linear-array transducer in musculoskeletal mode. For sternocleidomastoid assessment, participants will lie supine with the cervical spine in a neutral position and the neck muscles relaxed. For upper trapezius assessment, participants will be positioned prone without a pillow, with the hands placed beneath the forehead to promote relaxation of the posterior cervical region. Sternocleidomastoid measurements will be obtained at the midpoint between the sternal head and the mastoid process, and upper trapezius measurements will be obtained 2 cm lateral to the C4 spinous process. After acquisition of a stable grayscale image, the system will be switched to shear-wave elastography mode. A region of interest covering the target muscle will be selected, and Young’s modulus (kPa) will be recorded. Three measurements will be obtained on each side and averaged for analysis. All examinations will be performed by a trained assessor using a predefined standardized scanning protocol [29,30].

#### Secondary outcomes.

(1) bilateral sternocleidomastoid and upper trapezius SWE parameters;
(2) pain intensity measured by the Faces Pain Scale-Revised (FPS-R);
(3) cervical disability measured by the Pediatric Neck Disability Index (Pediatric NDI);
(4) cervical rotation range of motion;
(5) recurrence-related outcomes at 3 and 6 months after treatment;
(6) overall clinical efficacy at week 4.

#### Shear-wave elastography parameters.

SWE will assess the bilateral sternocleidomastoid and upper trapezius muscles at baseline, week 2, and week 4. These data will describe broader changes in cervical muscle mechanics during recovery and will support the interpretation of the primary outcome [30,31].

#### Faces pain scale-revised.

Pain intensity will be measured with the Faces Pain Scale-Revised, which is a validated self-report scale widely used in children. Scores range from 0 to 10, and higher scores indicate more severe pain. Pain will be assessed at baseline, week 2, week 4, and at 3 and 6 months after treatment.

#### Pediatric neck disability index.

Cervical disability will be measured with the Pediatric Neck Disability Index. The same language version and scoring method will be used at all assessment time points. A guardian may help the child understand the questions when needed, but the child will provide the response whenever possible. This outcome will be assessed at baseline, week 2, week 4, and at 3 and 6 months after treatment.

#### Cervical rotation range of motion.

Active cervical rotation range of motion on both sides will be measured using a standardized clinical goniometric method while the participant sits in a consistent position. Two measurements will be obtained on each side, and the average value will be used for analysis. This outcome will be assessed at baseline, week 2, and week 4.

#### Clinical efficacy evaluation.

Overall clinical efficacy will be evaluated at week 4 according to standardized criteria derived from the Diagnostic and Therapeutic Criteria for Traditional Chinese Orthopedic Diseases and the Criteria for Diagnosis and Evaluation of Clinical Disease Outcomes. Treatment response will be classified into three categories: (1) Recovered: complete resolution of pain, normal cervical appearance, normal cervical range of motion, and no impact on daily life; (2) Improved: marked reduction in pain, no obvious cervical tilt, mild limitation of cervical motion, and minimal impact on daily life; (3) Ineffective: slight reduction in pain, persistent obvious cervical tilt, significant limitation of cervical motion, and noticeable impact on daily life.

The overall treatment effectiveness rate will be calculated as follows:


$$\begin{array}{c} Effectiveness\text{\textasciitilde{}}rate\left(\%\right)=(number\text{\textasciitilde{}}of\text{\textasciitilde{}}recovered\text{\textasciitilde{}}cases+number\text{\textasciitilde{}}of\text{\textasciitilde{}}improved\text{\textasciitilde{}}cases)/total\text{\textasciitilde{}}number\text{\textasciitilde{}}of\text{\textasciitilde{}}participants\times 100\%\text{.} \end{array}$$

#### Recurrence-related outcomes.

Recurrence-related outcomes will be assessed at 3 and 6 months after treatment. Recurrence will be defined as the return of painful torticollis and limited cervical rotation that leads to additional medical evaluation or treatment during follow-up. Investigators will collect this information during follow-up contact and review of available medical records.

### Trial oversight

This is a single-center investigator-initiated trial coordinated by the Department of Tuina, Hangzhou Hospital of Traditional Chinese Medicine. The study team will be responsible for participant screening, recruitment, intervention delivery, outcome assessment, data collection and study coordination. The principal investigator will have overall responsibility for trial conduct and protocol compliance. No separate steering committee or endpoint adjudication committee has been established because this is a single-center trial with a modest sample size and low-risk physical interventions. Data management and quality control will be conducted by the study investigators according to predefined study procedures.

#### Trial monitoring.

Trial conduct will be monitored by the study investigators throughout the recruitment, intervention, follow-up, and data-management phases. Screening logs, recruitment progress, intervention adherence records, case report forms, protocol deviations, and adverse events will be reviewed at regular intervals, at least once every month during active recruitment and treatment. Source data checks and review of data completeness and consistency will be performed periodically by designated members of the study team who are not responsible for the participant’s routine treatment on the day of review, where feasible. Important protocol deviations, safety concerns, and data-quality issues will be documented and discussed by the study team, and corrective actions will be implemented when needed. No separate external trial monitoring committee has been established because this is a single-center study of low-risk physical interventions.

#### Safety monitoring.

All adverse events will be recorded during the intervention and follow-up periods. Adverse events will be assessed systematically at each treatment session and follow-up contact and will be classified by severity and relatedness to the intervention. Expected mild events may include temporary neck soreness, brief discomfort during exercise, or transient dizziness. The allocated intervention will be stopped if a participant develops intolerable symptoms, marked worsening of neurological signs, a serious adverse event or another condition requiring alternative medical management. Serious adverse events will be reported promptly to the ethics committee in accordance with local requirements.

#### Data monitoring committee.

A separate data monitoring committee will not be established for this trial because this is a single-center study of low-risk physical interventions with a modest sample size and no planned interim efficacy analyses. Trial conduct and safety oversight will instead be managed by the study investigators in accordance with the protocol and institutional requirements.

#### Interim analyses and stopping guidelines.

No formal interim efficacy analyses are planned because this is a single-center trial with a modest sample size and a relatively short intervention period. The study team will review safety events throughout the trial. The principal investigator will have access to accumulating safety information and will make the final decision on trial suspension or termination if there are significant safety concerns, major protocol problems, or ethical reasons requiring early discontinuation. Serious adverse events will be reported promptly to the ethics committee.

### Sample size

The sample size was calculated based on the primary outcome, namely affected-side sternocleidomastoid stiffness at week 4. Because directly comparable shear-wave elastography data from children with AARS were unavailable, the assumptions were informed by preliminary observations and published studies of cervical muscle stiffness measured using shear-wave elastography [25,26]. A target between-group difference of 6.5 kPa and a common standard deviation of 9.0 kPa were assumed, corresponding to a standardized effect size of approximately 0.72. With a two-sided significance level of 0.05, 80% power, and a 1:1 allocation ratio, 31 participants were required in each group. Allowing for approximately 10% attrition or unavailable week-4 primary outcome data, the final target sample size was set at 70 participants, with 35 participants in each group.

Given the limited pediatric AARS-specific elastography evidence available when the trial was designed, these assumptions should be interpreted cautiously. The trial is designed to test the prespecified primary hypothesis and will also provide estimates of treatment effects and outcome variability for future larger multicenter studies.

### Data collection, management and quality control

Trained investigators will record study data in standardized case report forms and then enter them into a password-protected electronic database. Each participant will be assigned a unique study identification number, and personally identifiable information will be stored separately from study data. Key outcome data will be entered independently by two trained staff members. Discrepancies will be checked against the original case report forms and source documents and resolved before database lock. Range checks and logical consistency checks will be performed regularly for demographic variables, eligibility criteria, treatment attendance, outcome scores, ultrasound values, adverse events, protocol deviations, and follow-up data. Data queries will be generated when missing, inconsistent, or implausible values are identified. These queries will be reviewed and resolved by authorized study personnel using source documents whenever possible. All corrections will be documented. Only authorized study staff will have access to the final dataset. Regular data-quality checks will be performed throughout recruitment, treatment, follow-up, and database preparation.

### Retention strategies

To minimize missing data and loss to follow-up, several retention strategies will be implemented. Appointment times will be scheduled flexibly according to the availability of the child and legal guardian. Guardians will receive reminders before treatment and assessment visits by telephone or electronic message. Missed visits will be followed up within 24–48 hours, and outcome assessments will be rescheduled as soon as possible within the prespecified assessment window. The study team will maintain regular contact with guardians during the intervention and follow-up periods to encourage treatment attendance, adherence to study instructions, and completion of outcome assessments. Reasons for missed treatment sessions, missed assessments, withdrawal, or loss to follow-up will be recorded whenever possible.

### Confidentiality

Personal information obtained during screening and trial participation will be collected only by authorized study staff and will be handled in a confidential manner. Participants will be assigned unique study identification numbers, and identifiable information will be stored separately from research data in secure files or password-protected electronic systems with restricted access. Paper records will be kept in locked storage areas at the study site, and electronic data will be stored on secure institutional systems. No directly identifiable participant information will be used in reports, presentations, or publications. Access to identifiable data will be limited to personnel who require such access for trial conduct, oversight, or regulatory purposes. After trial completion, study records will be retained and managed in accordance with applicable institutional and regulatory requirements.

### Missing data

Missing data are expected to be limited, with an anticipated missing data rate of approximately 10%, consistent with the dropout allowance used in the sample size calculation. When missing or inconsistent data are identified, the study team will first verify the original case report forms and source documents. Data queries will be generated and resolved by trained study personnel before database lock. If data remain missing, the reason for missingness will be documented whenever possible, including withdrawal, missed assessment, poor ultrasound image quality, inability to complete the assessment, receipt of prohibited co-interventions, or loss to follow-up.

The primary analysis will follow the intention-to-treat principle, with participants analyzed according to their originally assigned groups. The primary ANCOVA will use participants with available baseline and week-4 primary outcome data. If missing primary outcome data are considered likely to influence the results, multiple imputation under the missing-at-random assumption will be performed as a sensitivity analysis. Linear mixed-effects models for repeated secondary outcomes will use all available data under the missing-at-random assumption. No imputation will be performed for adverse events; these will be summarized based on observed data.

### Statistical analysis

Statistical analyses will be performed using SPSS version 22.0. Continuous variables will be presented as mean ± standard deviation when normally distributed or as median (interquartile range) when not normally distributed. Categorical variables will be presented as frequencies and percentages.

The statistical analysis plan was refined in this manuscript to meet randomized trial reporting standards. The original ethics-approved protocol specified descriptive statistics and pre-post comparisons. The manuscript further specifies baseline-adjusted between-group analysis for the primary endpoint and repeated-measures models for longitudinal outcomes.

The intention-to-treat population will include all randomized participants, who will be analyzed according to their originally assigned groups. The primary ANCOVA will be based on participants with available baseline and week-4 primary outcome data. Multiple imputation under the missing-at-random assumption will be performed as a sensitivity analysis if missing primary outcome data are considered likely to influence the results.

The primary outcome will be analyzed with analysis of covariance, with affected-side sternocleidomastoid stiffness at week 4 as the dependent variable, treatment group as the fixed factor, and baseline affected-side sternocleidomastoid stiffness as the covariate. Adjusted between-group differences with 95% confidence intervals will be reported. To avoid overfitting given the modest sample size, additional covariate adjustment will be limited to prespecified clinically relevant variables. A supplementary adjusted model may include age, sex, baseline symptom duration, and baseline pain intensity, if model assumptions and data completeness permit. These analyses will be considered supportive of the primary baseline-adjusted analysis.

Repeated secondary outcomes will be analyzed according to their scheduled assessment time points. SWE parameters and cervical rotation range of motion will be assessed at baseline, week 2, and week 4. Pain intensity and cervical disability will be assessed at baseline, week 2, week 4, and at 3 and 6 months after treatment. Recurrence-related outcomes will be assessed at 3 and 6 months after treatment. Linear mixed-effects models will be used for repeated outcomes, with terms for group, time, and the group-by-time interaction, where appropriate. The overall effectiveness rate at week 4 will be compared between groups using the chi-square test or Fisher’s exact test, as appropriate.

The primary outcome will be tested at a two-sided significance level of 0.05 without adjustment for multiplicity. Secondary outcomes will be interpreted as supportive and exploratory. To address potential multiple comparison issues across secondary outcomes, adjusted P values using the Holm-Bonferroni method will be reported where appropriate, together with effect estimates and 95% confidence intervals. Interpretation of secondary outcomes will emphasize the magnitude and precision of treatment effects rather than statistical significance alone.

Model assumptions will be assessed before final interpretation of the primary and secondary analyses. For the ANCOVA model, residual distribution, homogeneity of variance, linearity between the baseline covariate and the week-4 outcome, and influential observations will be examined using residual plots and appropriate diagnostic procedures. For linear mixed-effects models, residual distribution, variance structure, and model convergence will be assessed. If substantial deviations from model assumptions are observed, data transformation or robust standard errors will be considered where appropriate. For the primary outcome, a rank-based ANCOVA adjusted for the baseline value of the primary outcome will be used as a non-parametric sensitivity analysis if the assumptions of the standard ANCOVA are not met. For repeated secondary outcomes, if the assumptions of linear mixed-effects models are seriously violated, transformed outcome models, robust variance estimation, or rank-based repeated-measures approaches will be considered as sensitivity analyses. These alternative analyses will be interpreted as sensitivity analyses, while the prespecified ANCOVA and linear mixed-effects models will remain the primary analytical framework.

Binary recurrence-related outcomes at the 3- and 6-month follow-up visits will be compared using the chi-square test or Fisher’s exact test, as appropriate. Additional exploratory analyses may be conducted for other ultrasound-derived measures according to the final data structure and distribution.

### Sensitivity analyses

The intention-to-treat analysis will be considered the primary analysis. A per-protocol analysis will be performed as a sensitivity analysis to assess the robustness of the primary findings. The per-protocol population will include participants who complete the week-4 primary outcome assessment, receive at least 80% of the assigned treatment sessions, have no major protocol deviations, and do not receive prohibited co-interventions during the intervention period. Sensitivity will be evaluated by comparing the direction, magnitude, and 95% confidence intervals of the treatment effect between the intention-to-treat and per-protocol analyses. If the results are consistent across these analyses, the findings will be considered robust. If meaningful discrepancies are observed, the potential influence of adherence, missing data, and protocol deviations will be examined and reported.

All tests will be two-sided. A P value <0.05 will be considered statistically significant for the primary analysis. Secondary analyses will be interpreted as supportive and exploratory, with multiplicity-adjusted P values reported where appropriate.

### Patient and public involvement

Patients and members of the public were not formally involved in the design, conduct, reporting, or dissemination planning of this protocol. The intervention schedule and outcome assessment procedures were developed by the clinical and research team on the basis of clinical experience, feasibility, and the published literature. A plain language summary of the study findings will be made available to participating children and their legal guardians on request after completion of the trial.

### Trial status

The ethics-approved Chinese protocol is version 2.0. The submitted English protocol manuscript was prepared as a reporting version based on the ethics-approved protocol. The trial was registered in the International Traditional Medicine Clinical Trial Registry (ITMCTR2025002448) on 27/10/2025. Recruitment began on 27/10/2025 after trial registration had been completed. No participant was enrolled before completion of trial registration. Recruitment is expected to be completed on 31/10/2026. Because recurrence-related outcomes will be assessed at the 6-month follow-up after treatment, data collection is expected to be completed by 31/05/2027.

### Protocol and statistical analysis plan access

The original ethics-approved clinical trial protocol in Chinese is provided as S1 File, and its complete English translation is provided as S2 File. A detailed statistical analysis plan will be finalized, dated, and retained by the investigators before database lock and before the main analysis is performed. The statistical analysis plan will be made available on reasonable request, subject to applicable ethical and institutional requirements.

## Discussion

This trial will evaluate whether adding cervical resistance training to joint mobilization provides greater benefit than joint mobilization alone in children with AARS. The study addresses an important gap in pediatric cervical rehabilitation, because conservative treatment is widely used in clinical practice but structured high-quality randomized evidence remains limited [1,3,8]. The trial also reflects the current emphasis on early conservative management, which is supported by previous pediatric series showing that prompt diagnosis and timely nonsurgical treatment may improve outcomes [30–32]. At the same time, recent reviews indicate that the optimal nonsurgical strategy for pediatric AARS has not yet been clearly established and that delayed presentation may increase the risk of relapse, treatment failure, or subsequent surgical management [33–35].

This protocol has several strengths, including its randomized controlled design, blinded outcome assessment, and a prespecified primary analysis approach. It also uses a single primary imaging endpoint, namely affected-side sternocleidomastoid stiffness at week 4, which may improve interpretability and reduce outcome ambiguity. In addition, musculoskeletal ultrasound and shear-wave elastography provide objective outcome measures and complement standard clinical assessments of pain, disability and range of motion. The detailed intervention protocol may also support reproducibility in future studies and clinical practice.

This protocol also has several limitations. Full blinding is not possible because the two interventions are clearly different. Therapist contact time is greater in the intervention group than in the control group, which may influence non-specific treatment effects. In addition, the trial is conducted at a single center, which may limit generalizability. Ultrasound-based muscle assessment is also operator-dependent, although a predefined standardized scanning protocol will be used. Finally, adherence may vary across participants, particularly with respect to attendance at supervised sessions and compliance with study instructions outside treatment sessions.

Even with these limitations, the trial is expected to provide clinically useful evidence on muscle-focused conservative treatment for pediatric AARS. The findings may also clarify whether ultrasound-based muscle markers can help monitor recovery and guide rehabilitation decisions in this population.

## Supporting information

S1 File Original ethics-approved Chinese protocol. (PDF)

S2 File Complete English translation of the original ethics-approved clinical trial protocol. (DOCX)

S3 File SPIRIT checklist. (DOCX)
