# Supplementary material for: Joint mobilization combined with cervical resistance training for pediatric atlantoaxial rotatory subluxation: Protocol for a randomized controlled trial with musculoskeletal ultrasound assessment
Source: PLoS One. 2026 Sep 24;21(9):e0359198. doi: 10.1371/journal.pone.0359198 (PMC13606960; doi:10.1371/journal.pone.0359198)
Supplement: Supplementary file 1 [file pone.0359198.s001.pdf]

科研课题伦理申请临床研究方案

项目名称	通过 SWE 探索以关节松动术为基础的多维度抗阻训练治疗儿童寰枢关节半脱位的临床研究		
课题编号	2024KY1378	立项部门	浙江省医药卫生科技计划
负 责 人	蔡阁	邮 箱	365727190@qq.com
科 室	推拿科	联系电话	15397149327
研究方案	（一）研究背景：<br>寰枢关节旋转性移位又称为寰枢关节半脱位（atlanto-axial rotatory subluxation, AARS）高发于儿童少年期。临床表现多为颈项部疼痛、僵硬、歪斜、旋转，不能自动或被动矫正，严重脱位者或伴头晕、恶心、心悸、走路不稳等神经、血管、脊髓功能障碍。随着学习压力增大及电子设备使用率增高，我国儿童寰枢关节半脱位发生率呈上升趋势，寰枢关节半脱位所造成的症状类似于短暂性良性斜颈，若未得到有效治疗，易出现长期颈部歪斜、疼痛等并发症，甚至可能导致永久性伤残及死亡，患儿症状反复出现，不仅影响患儿体态，而且易造成心理及精神压力，在这种情况下，寻找一种经济、便捷、安全、疗效持久显著的治疗方案至关重要。<br>（二）研究目标：<br>通过超声弹性成像技术明确多维抗阻训练结合关节松动术治疗儿童寰枢关节半脱位的临床疗效及安全性，证明以关节松动术为基础的多维抗阻训练在儿童寰枢关节半脱位治疗中的促进作用，为临床治疗儿童寰枢关节半脱位提供参考依据。<br>（三）计划纳入病例数： 70 人<br>（四）研究中心数： 1 个<br>（五）收集病例时间：2024 年 3 月—— 2025 年 12 月<br>（六）研究时间：取得伦理审查批件之后开始研究，到 2026 年 12 月结束。		

研究对象：

收集 70 例经专业儿科、骨科医生明确诊断，且符合诊断标准儿童寰枢关节半脱位患者，年龄范围 2-18 岁。

纳排标准：

1、诊断标准：

参考《实用骨科学》和临床经验制定。

（1）病史：近期多有上呼吸道感染、颈部淋巴结炎、中耳炎等和（或）颈项部外伤史和（或）长期伏案、低头、姿势不良史；

（2）症状：颈项部疼痛、僵硬、歪斜、旋转，不能自动或被动矫正；偶伴头晕、头痛、胸闷、心悸等不适；

（3）查体：颈椎低头、仰头及旋转活动受限 ，上颈段肌肉紧 张痉挛，触之有胀厚感及筋结，在枢椎、椎板或横突处均有明显压痛，并有高于对侧的隆起感或棘突偏移；

（4）颈椎正侧位、寰枢椎张口位或 CT 提示：颈椎曲度变直或反弓、颈椎侧弯并有椎体旋转、寰枢关节不对称、寰椎双边征、寰枢边缘阶梯状等（侧位片寰齿前间距＞4 mm；张口位枢椎齿状突与寰椎侧块间距离不对称，寰齿间隙差值＞3 mm，寰枢关节外侧缘不齐，侧块移位＞2 mm。CT 表现：冠状位寰椎侧块两侧下角连线中点的垂直线，与齿状突轴线偏移>2-3 mm；齿状突侧缘与寰椎侧块内缘间距双侧不等宽（>2-3 mm）；寰椎与枢椎椎体两侧缘发生同向不连续）。

2、纳入标准：

（1）符合寰枢关节半脱位诊断标准；

（2）年龄在 2-18 岁之间；

（3）无其他严重疾病，且能积极配合治疗和检查；

（4）签署本研究知情同意书。

3、排除标准：

（1）不符合寰枢关节半脱位诊断的其他颈痛伴活动受限患者，如落枕、肌性斜颈、结核、肿瘤、骨折等；

（2）符合寰枢关节半脱位诊断，但年龄不在规定范畴、合并其他严重疾患、不能积极配合治疗或检查、正在接受其他治疗方案、病情严重且不适合保守治疗者、资料不全者。

研究内容：

收集 70 例经专业儿科、骨科医生明确诊断，且符合诊断标准儿童寰枢关节半脱位患者，年龄范围 2-18 岁。所有入组患者随机分为关节松动术对照组和多维度抗阻训练结合关节松动术治疗组。2 组患者治疗前后采用 NDI 颈部功能症状评分及 VAS 评分表进行功能症状评分，颈椎活动度，颈部肌肉(斜方肌、头夹肌、胸锁乳突肌)肌肉组织弹性，并评价临床疗效。

研究方法和技术路线：

1、研究方法：

本研究用随机对照研究方法，将符合标准的 70 例患者随机分为 2 组，每组各 35 例，采用一个临床试验中心进行。运用 SPSS(22.0 版)软件自编宏程序进行随机分组。当有符合纳入标准的患者纳入研究时，按其进入试验的先后顺序，并根据随机分组结果入组。

1 干预方案

1.1 对照组（关节松动术）

对照组施以关节松动术，具体操作：（1）寰枢关节分离牵引松动：患者仰卧位，治疗师一手虎口置于患者后枕部，另一手置于患者下颌部，双手在牵引基础上颈部做屈伸、侧屈、旋转动作，每个动作操作 5 次，每次持续 6s 左右的牵张。（2）静态关节松动：患者取坐位，治疗师自后向前规律性按压棘突，节律性按压颈椎两侧横突，椎间关节垂直松动，棘突侧方按推，每个动作操作 5 次，每次持续 6s 左右的牵张。（3）动态关节松动术：患者取坐位，颈稍前倾，治疗师右手食指 、中指 、无名指托住枕骨，小指中段靠在第 2 颈椎棘突，左手大鱼际压在右手小指上，轻轻地将第 2 颈椎往腹侧推，此时枕骨被医者的右手臂固定。第 2 颈椎的椎体先往腹侧滑动，沿椎体小关节斜向上进行节律性关节松动，当第 2 颈椎往前推到底时，第 1 颈椎也开始往前滑动，以小范围节律性被动活动为主力，作用于下一节段椎体小关节，产生一个斜向上的滑动，使上一节段椎体小关节相对于下方产生一个斜向上的松动效果，当第 1 、2 颈椎往前滑到底时至少要 停留 10s。手法等级以病人耐受为宜。

1.2 治疗组（多维度抗阻训练结合关节松动术）

1.2.1 关节松动术：操作同对照组

1.2.2 颈部肌群单一维度抗阻训练：①前屈：患者双手交叉重叠置于前额，手掌施力阻抗头部前屈动作，保持头部平衡不移动；②后伸：患者双手交叉重叠置于枕部，手掌施力阻抗头部后伸动作，保持头部平衡不移动；③左右旋转：双手分别置于头部颞侧，一侧手掌施力阻抗患者转头看肩膀动作，保持头部平衡不移动，左右旋转依次进行；④左、右侧屈：双手分别置于头部颞侧，一侧手掌施力阻抗患者颈部侧屈使耳朵贴近肩部动作，尽量保持头部平衡不移动，左右旋转依次进行；⑤中立位：颈部维持中立位，手掌分别从面颊、后枕、前额等处分别施力，尽量保持颈部维持中立位不移动。每个动作维持 10 s，练习 10 次为 1 组，每天练习 3 组。

1.2.3 颈部肌群多维度抗阻训练：①前屈抗阻下做左右旋转运动：患者仰卧位，头部悬空，治疗师徒手放在患者前额部，并施加使头颈部向后运动的力。施力的同时，患者头颈部做前屈动作，前屈动作力和手掌施力作用相反。患者在前屈抗阻训练中，努力保持头颈部的平衡同时，做左右转头看肩膀旋转运动；②前屈抗阻下左右侧屈动作：患者体位参照第一个动作，患者在前屈抗阻训练中，努力保持头颈部的平衡同时，做左右侧屈使耳朵贴近肩部动作；③后伸抗阻下做左右旋转运动：患者俯卧位，头部悬空，治疗师徒手放在患者后枕部，并施加使头颈部向前运动的力。施力的同时，患者头颈部做后伸动作，后伸动作力和手掌施力作用相反。患者在后伸抗阻训练中，努力保持头颈部的平衡同时，做左右转头看肩膀旋转运动；④后伸抗阻下做左右侧屈运动：患者体位参照第三个动作，患者在后伸抗阻训练中，努力保持头颈部的平衡同时，做左右侧屈使耳朵贴近肩部动作。上述动作练习 15 次为 1 组，每天练习 3 组。

1.3 疗程：隔天 1 次，每周 3 次（周一、三、五），周末双休，连续治疗 4 周（共 12 次）

1.4 随访：于治疗结束后的 3 个月、6 个月采用门诊复诊、电话、电子邮件、家访

等方式对受试者进行随访。随访指标:VAS 疼痛评分、NDI 评分。

1.5 观察指标:

1.5.1 观察时间:评估选取的时间点为治疗前、治疗结束(第 12 次治疗后)。治疗结束后的 3 个月、6 个月为随访观察时间点。

1.5.2 观察项目

1.5.2.1 一般项目：包括性别、年龄、病程、病史、病因等

1.5.2.2 疼痛强度:采用疼痛视觉模拟评分法(visual analogue scale, VAS):用一条约长 10cm 的标尺，一面刻有 10 个刻度，两端分别为 “0”和“10”。“0”表示无痛，“10”表示剧烈疼痛。分数越大，表示疼痛强度越强。以此评价颈部疼痛情况。

1.5.2.3 颈部功能症状评分：采用颈椎功能障碍指数量表（neck disability index,NDI）该量表共包括疼痛程度、个人生活料理、阅读、头痛、注意力、抬物、工作、驾驶、睡眠和娱乐 10 项内容，每项内容评分 0～5 分，NDI 总分 0（无障碍）～50 分（完全瘫痪），评分越高，患者颈椎功能障碍越严重。

1.5.2.4 颈椎活动度：根据《康复疗法评定学》,利用关节活动度量角器测量患者颈椎前屈、后伸、左右旋转、左右侧屈活动度。

1.5.2.5 颈部肌肉(斜方肌、头夹肌、胸锁乳突肌)肌肉组织弹性:分别于治疗前、后利用剪切波弹性成像技术(shear wave elastography，SWE)测定全部受试者颈部肌肉获取杨氏模量值(E 值)。超声设备选用法国产 Supersonic Aixplorer 超声诊断仪，15L 线阵探头，4-15MHz，选取肌肉-骨骼检查模式。

颈部肌肉 E 值测定：患者取俯卧位，双手交叠枕于额下，肩膀充分外展外旋，维持头颈静息中立位，使颈部肌肉放松。利用超声探头纵向定位，调节感兴趣区，确保完全覆盖斜方肌、胸锁乳突肌、头夹肌，待获得稳定、清晰的二维超声图像后切换至剪切波弹性成像模式，观察组织弹性图，系统自动计算出 Q-BOX 区域内肌肉组织的杨式模量值（E 值）。测量 3 次，取平均值。先左侧后右侧。

注:将采集到的图像和数据统一整理储存，数据统计时取左、右两侧数据均值。

1.6 临床疗效评估:参考《临床疾病诊断依据治愈好转标准》制定标准

痊愈:治疗后患儿疼痛消失,颈部外观正常，颈部活动正常，生活无影响;

有效:治疗后患儿疼痛明显减轻，颈部外观未见明显倾斜，颈部活动轻微受限，生活基本无影响;

无效:治疗后患儿疼痛稍减轻，颈部外观仍明显倾斜，颈部活动明显受限，对生活有一定影响。

有效率%=(痊愈+有效患者)例数/总患者例数

1.7 安全性和依从性评价

1.7.1 安全性:与治疗有关的不良事件例数。

1.7.2 依从性:两组入选病例中止和脱落病例的例数。

1.8 干预方案的分配方法

通过 SPSS 宏程序完成分配序列，将受试者就诊顺序与随机数字进行匹配并完成分组。将顺序号对应分组装入不透明信封内，完成受试者的干预分配。

1.9 数据收集、管理和分析方法

本研究采用统一病例报告表收集研究数据。所有受试者在签署知情同意书后，由经过统一培训的研究人员进行资料采集。基线资料包括性别、年龄、病程、发病诱因、既往病史、影像学检查结果、受累侧别及既往治疗情况。疗效资料包括疼痛评分、颈椎功能障碍指数评分、颈椎活动度、剪切波弹性成像测得的颈部目标肌肉杨氏模量值、临床疗效、安全性和依从性。治疗过程资料包括治疗日期、治疗完成情况、抗阻训练完成情

况、不良事件及处理情况。治疗结束后 3 个月和 6 个月进行随访，记录疼痛评分、颈椎功能障碍指数评分、症状复发情况及是否接受其他相关治疗。

剪切波弹性成像检查由接受统一培训的超声检查人员完成。检查时按照统一体位、统一测量部位和统一操作流程进行。每块目标肌肉每侧测量 3 次，取平均值作为最终分析值。所有超声图像和原始测量数据统一编号保存。评估人员应尽量避免了解受试者分组情况。

所有研究数据由专人管理。纸质病例报告表应完整、清晰、及时填写。电子数据采用双人独立录入。录入完成后由数据管理员进行核对。若发现不一致，应返回原始病例报告表进行核查。数据录入时进行逻辑检查和范围检查，包括年龄范围、评分范围、治疗次数、随访时间和超声测量值范围。受试者个人身份信息与研究数据分开保存。用于统计分析的数据集仅保留受试者编号、分组信息和研究变量。

研究过程中应详细记录中止、脱落和失访原因。所有完成随机分组的受试者均纳入意向性分析。完成规定治疗疗程、主要结局资料完整且无重大方案违背的受试者纳入符合方案分析。所有至少接受一次治疗的受试者纳入安全性分析。

1.10 统计方法

本研究采用 SPSS 22.0 软件进行统计分析。所有统计检验均采用双侧检验。$P < 0.05$ 表示差异具有统计学意义。计量资料先进行正态性检验。符合正态分布的数据采用均数 ± 标准差表示，组内治疗前后比较采用配对 t 检验，组间比较采用独立样本 t 检验。不符合正态分布的数据采用中位数和四分位数间距表示，组内比较采用 Wilcoxon 符号秩检验，组间比较采用 Mann-Whitney U 检验。计数资料采用例数和百分比表示，组间比较采用卡方检验或 Fisher 精确检验。

主要疗效指标为治疗结束时颈部目标肌肉剪切波弹性成像测得的杨氏模量值变化。主要分析比较两组治疗前后变化值的差异。必要时采用协方差分析，以治疗后数值为因变量，治疗组别为固定因素，基线数值为协变量。疼痛评分、颈椎功能障碍指数评分、颈椎活动度和临床疗效作为次要疗效指标进行分析。临床疗效分为痊愈、有效和无效，总有效率按“痊愈例数+有效例数”占总例数的百分比计算。安全性分析包括不良事件类型、发生率、严重程度和处理情况。依从性分析包括治疗完成率、随访完成率、中止和脱落情况。若主要结局存在缺失数据，应说明缺失原因，并进行敏感性分析。

杭州市中医院科研伦理委员会

伦理审查批件

**项目名称**	通过 SWE 探索以关节松动术为基础的多维度抗阻训练治疗儿童寰枢关节半脱位的临床研究	**批件号**	2024KLL088
**申请状态**	☑新方案　☐修订版　☐修正版	**审查类型**	☑常规　☐快速　☐紧急
**研究者**	蔡阁		
**类　别**	科研类		
**审查文件**	1.伦理申请表<br>2.主要研究者责任声明<br>3.研究者利益冲突声明<br>4.临床研究方案（版本号 2.0）<br>5.知情同意书（版本号 2.0）<br>6.免除知情同意申请书（ x ）<br>7.数据管理员保密协议（ x ）		
**注意事项**	1.请遵循我国相关法律、法规和规章中的伦理原则。<br>2.请遵循经本伦理审查委员会批准的临床研究方案、知情同意书、招募材料等开展本研究，保护受试者的健康与权利。对研究方案、知情同意书和招募材料等的任何修改，均须得到本伦理审查委员会审查同意后方可实施。<br>3.在杭州市中医院发生的 SAE/SUSAR 以及研发期间安全性更新报告须按照 NMPA/GCP 最新要求及时递交本伦理审查委员会，国内外其它中心发生的 SAE/SUSAR 需定期汇总、评估后递交本伦理审查委员会。<br>4.根据报告情况，本伦理审查委员会有权对其评估做出新的决定。<br>5.当出现任何可能显著影响研究进行或增加受试者危险的情况时，请申请人及时向本伦理审查委员会提交书面报告。<br>6.研究纳入了不符合纳入标准或符合排除标准的受试者，符合中止研究规定而未让受试者退出研究，给予错误治疗或剂量，给予方案禁止的合并用药等没有遵从方案开展研究的情况；或可能对受试者的权益或健康以及研究的科学性造成不良影响等违背 GCP 原则的情况，请研究者提交违背方案报告。<br>7.申请人暂停或提前终止临床研究，请及时提交暂停或终止研究报告。<br>8.完成临床研究，请申请人提交结题报告。<br>9.凡涉及中国人类遗传资源采集标本、收集数据等研究项目，必须获得中国人类遗传资源管理办公室批准后方可在本中心开展研究。<br>10.项目启动前，请前往**“医学研究登记备案信息系统”**完成项目备案。<br>11.本批件有效期**三年**，自签发之日算起。		
**审查意见**	☑同意，☐作必要的修正后同意，☐作必要的修正后重审，☐不同意，☐终止或暂停已批准的研究。		
**持续审查频率**	☐3 个月　☐6 个月　☑12 个月　☐其他		
**主任委员签名**		**日　期**	2024 年 3 月 20 日

联系方式：杭州市体育场路 453 号，电话：0571-85827896
