# Supplementary material for: Joint mobilization combined with cervical resistance training for pediatric atlantoaxial rotatory subluxation: Protocol for a randomized controlled trial with musculoskeletal ultrasound assessment
Source: PLoS One. 2026 Sep 24;21(9):e0359198. doi: 10.1371/journal.pone.0359198 (PMC13606960; doi:10.1371/journal.pone.0359198)
Supplement: Supplementary file 2 [file pone.0359198.s002.docx]

**Complete English Translation of the Original Ethics-Approved Chinese Clinical Trial Protocol**

**Protocol Version 2.0**

Project No. 2024KY1378

Ethics Approval No. 2024KLL088

*Translator's note: This document is a complete English translation of the original ethics-approved Chinese clinical trial protocol (Version 2.0) and the accompanying ethics approval document. It has been prepared for journal submission. In the event of any discrepancy, the original Chinese documents remain authoritative.*

Hangzhou Hospital of Traditional Chinese Medicine

Affiliated to Zhejiang Chinese Medical University

# Clinical Research Protocol for Ethics Application of a Scientific Research Project

| **Project title** | A Clinical Study Using Shear-Wave Elastography (SWE) to Explore Multidimensional Resistance Training Based on Joint Mobilization for the Treatment of Pediatric Atlantoaxial Rotatory Subluxation |
| --- | --- |
| **Project number** | 2024KY1378 |
| **Project approval department** | Zhejiang Provincial Medical and Health Science and Technology Program |
| **Principal investigator** | Ge Cai |
| **Email** | 365727190@qq.com |
| **Department** | Department of Tuina |
| **Telephone** | 15397149327 |

# Study Protocol

## 1. Research Background

Atlantoaxial rotatory displacement, also known as atlantoaxial rotatory subluxation (AARS), occurs frequently during childhood and adolescence. Clinical manifestations commonly include neck pain, stiffness, torticollis, and rotational deformity that cannot be corrected actively or passively. Severe subluxation may be accompanied by dizziness, nausea, palpitations, unsteady gait, and other neurological, vascular, or spinal cord dysfunction. With increasing academic pressure and the growing use of electronic devices, the incidence of pediatric atlantoaxial rotatory subluxation in China has shown an increasing trend. The symptoms caused by atlantoaxial rotatory subluxation may resemble transient benign torticollis. Without effective treatment, long-term neck tilt, pain, and other complications may occur, and severe cases may even lead to permanent disability or death. Recurrent symptoms not only affect the child's posture but may also cause psychological and emotional stress. Therefore, it is important to identify an economical, convenient, safe, durable, and effective treatment strategy.

## 2. Research Objective

This study will use ultrasound elastography to determine the clinical efficacy and safety of multidimensional resistance training combined with joint mobilization for pediatric atlantoaxial rotatory subluxation. The study aims to demonstrate the additional therapeutic effect of multidimensional resistance training based on joint mobilization and to provide a reference for the clinical treatment of pediatric atlantoaxial rotatory subluxation.

## 3. Planned Sample Size

A total of 70 participants will be enrolled.

## 4. Number of Study Centers

One study center.

## 5. Case Collection Period

March 2024 to December 2025.

## 6. Study Period

The study will begin after ethics approval is obtained and will end in December 2026.

# Study Participants

A total of 70 pediatric patients with atlantoaxial rotatory subluxation will be recruited. Each patient will have a definite diagnosis made by professional pediatricians and orthopedic physicians and will meet the diagnostic criteria. The age range will be 2-18 years.

# Eligibility Criteria

## 1. Diagnostic Criteria

The diagnostic criteria were formulated with reference to Practical Orthopedics and clinical experience.

**(1)** Medical history: A recent history of upper respiratory tract infection, cervical lymphadenitis, otitis media, and/or cervical trauma, and/or a history of prolonged desk work, sustained head-down posture, or poor posture.

**(2)** Symptoms: Neck pain, stiffness, torticollis, and rotational deformity that cannot be corrected actively or passively; occasional dizziness, headache, chest tightness, palpitations, or other discomfort.

**(3)** Physical examination: Limited cervical flexion, extension, and rotation; tension and spasm of the upper cervical muscles; a swollen or thickened sensation and palpable nodules; marked tenderness over the axis, lamina, or transverse process; and a prominence higher than the contralateral side or deviation of the spinous process.

**(4)** Imaging findings: Anteroposterior and lateral cervical radiographs, open-mouth atlantoaxial radiographs, or computed tomography may show straightening or reversal of the cervical curvature, cervical scoliosis with vertebral rotation, atlantoaxial joint asymmetry, a double-border sign of the atlas, or step-like changes at the atlantoaxial margins. On lateral radiographs, the anterior atlantodental interval is greater than 4 mm. On open-mouth radiographs, the distance between the odontoid process of the axis and the lateral masses of the atlas is asymmetric, with a difference in the atlanto-odontoid spaces greater than 3 mm; the lateral margins of the atlantoaxial joints are irregular; or lateral mass displacement is greater than 2 mm. Computed tomography may show that, on coronal images, the vertical line through the midpoint of the line connecting the lower corners of the bilateral atlas lateral masses deviates from the axis of the odontoid process by more than 2-3 mm; the distances between the lateral margins of the odontoid process and the medial margins of the atlas lateral masses are unequal bilaterally by more than 2-3 mm; or the bilateral margins of the atlas and axis vertebral bodies show discontinuity in the same direction.

## 2. Inclusion Criteria

**(1)** Meeting the diagnostic criteria for atlantoaxial rotatory subluxation.

**(2)** Age between 2 and 18 years.

**(3)** No other serious disease and able to cooperate actively with treatment and examinations.

**(4)** Signed informed consent for participation in this study.

## 3. Exclusion Criteria

**(1)** Other causes of neck pain with restricted motion that do not meet the diagnostic criteria for atlantoaxial rotatory subluxation, such as acute stiff neck, muscular torticollis, tuberculosis, tumor, or fracture.

**(2)** Patients who meet the diagnostic criteria for atlantoaxial rotatory subluxation but are outside the specified age range; have other serious diseases; cannot cooperate actively with treatment or examinations; are currently receiving another treatment program; have severe disease and are unsuitable for conservative treatment; or have incomplete data.

# Study Content

A total of 70 pediatric patients with atlantoaxial rotatory subluxation will be recruited after definite diagnosis by professional pediatricians and orthopedic physicians and confirmation that they meet the diagnostic criteria. The age range will be 2-18 years. All enrolled patients will be randomly assigned to a joint mobilization control group or a multidimensional resistance training combined with joint mobilization treatment group. Before and after treatment, both groups will be assessed using the Neck Disability Index (NDI), the Visual Analogue Scale (VAS), cervical range of motion, the tissue elasticity of the neck muscles (trapezius, splenius capitis, and sternocleidomastoid), and clinical efficacy.

# Research Methods and Technical Route

## 1. Research Method

This study will use a randomized controlled design. Seventy eligible patients will be randomly assigned to two groups, with 35 participants in each group, at a single clinical trial center. Randomization will be performed using a self-programmed macro in SPSS software (version 22.0). When an eligible patient is enrolled, the patient will be assigned according to the order of entry into the trial and the randomization result.

## 1. Intervention Protocol

### 1.1 Control Group: Joint Mobilization

The control group will receive joint mobilization. The specific procedures are as follows.

**(1)** Atlantoaxial separation traction mobilization: The patient will be placed in the supine position. The therapist will place the web space of one hand at the posterior occipital region and the other hand at the mandible. While maintaining traction with both hands, the therapist will move the neck into flexion and extension, lateral flexion, and rotation. Each movement will be performed 5 times, and each stretch will last approximately 6 seconds.

**(2)** Static joint mobilization: The patient will be placed in the sitting position. The therapist will apply regular posterior-to-anterior pressure to the spinous processes, rhythmic pressure to the transverse processes on both sides of the cervical spine, vertical mobilization of the intervertebral joints, and lateral pushing and pressing of the spinous processes. Each maneuver will be performed 5 times, and each stretch will last approximately 6 seconds.

**(3)** Dynamic joint mobilization: The patient will be seated with the neck slightly flexed. The therapist will support the occiput with the index, middle, and ring fingers of the right hand, place the middle segment of the little finger against the C2 spinous process, and press the thenar eminence of the left hand onto the right little finger to gently push C2 anteriorly. The occiput will be stabilized by the therapist's right forearm. The C2 vertebral body will first glide anteriorly, and rhythmic joint mobilization will be applied obliquely upward along the facet joint. When C2 reaches the end of its anterior glide, C1 will also begin to glide anteriorly. Small-amplitude rhythmic passive movement will be applied to the facet joint of the adjacent segment to produce an oblique upward glide, allowing the upper-segment facet joint to mobilize obliquely upward relative to the lower segment. When C1 and C2 reach the end of the anterior glide, the position will be maintained for at least 10 seconds. The grade of mobilization will be adjusted according to patient tolerance.

### 1.2 Treatment Group: Multidimensional Resistance Training Combined with Joint Mobilization

### 1.2.1 Joint Mobilization

The procedure will be the same as that used in the control group.

### 1.2.2 Single-Dimensional Resistance Training of the Cervical Muscle Groups

**(1)** Flexion: The patient will place both hands crossed and overlapped on the forehead. The palms will apply force to resist head flexion while the head is kept balanced and does not move.

**(2)** Extension: The patient will place both hands crossed and overlapped on the occipital region. The palms will apply force to resist head extension while the head is kept balanced and does not move.

**(3)** Left and right rotation: The hands will be placed on the temporal regions of the head. One palm will apply force to resist the movement of turning the head toward the shoulder while the head is kept balanced and does not move. Left and right rotation will be performed sequentially.

**(4)** Left and right lateral flexion: The hands will be placed on the temporal regions of the head. One palm will apply force to resist cervical lateral flexion toward the shoulder while the head is kept as balanced and still as possible. The left and right sides will be performed sequentially.

**(5)** Neutral position: The neck will be maintained in a neutral position. The palms will apply force separately from the cheek, posterior occiput, forehead, and other positions while the neck is kept in a neutral position and as still as possible. Each movement will be maintained for 10 seconds. Ten repetitions will constitute 1 set, and 3 sets will be practiced each day.

### 1.2.3 Multidimensional Resistance Training of the Cervical Muscle Groups

**(1)** Left and right rotation under flexion resistance: The patient will be placed in the supine position with the head unsupported. The therapist will place a hand on the patient's forehead and apply a force directed toward head and neck extension. At the same time, the patient will perform head and neck flexion against the force applied by the therapist. While maintaining balance of the head and neck during flexion resistance, the patient will rotate the head left and right to look toward the shoulder.

**(2)** Left and right lateral flexion under flexion resistance: The patient's position will be the same as in the first movement. While maintaining balance of the head and neck during flexion resistance, the patient will perform left and right lateral flexion to bring the ear toward the shoulder.

**(3)** Left and right rotation under extension resistance: The patient will be placed in the prone position with the head unsupported. The therapist will place a hand on the posterior occiput and apply a force directed toward head and neck flexion. At the same time, the patient will perform head and neck extension against the force applied by the therapist. While maintaining balance of the head and neck during extension resistance, the patient will rotate the head left and right to look toward the shoulder.

**(4)** Left and right lateral flexion under extension resistance: The patient's position will be the same as in the third movement. While maintaining balance of the head and neck during extension resistance, the patient will perform left and right lateral flexion to bring the ear toward the shoulder. The above movements will be practiced for 15 repetitions as 1 set, and 3 sets will be practiced each day.

### 1.3 Treatment Course

Treatment will be performed once every other day, 3 times per week (Monday, Wednesday, and Friday), with no treatment on weekends, for 4 consecutive weeks, for a total of 12 sessions.

### 1.4 Follow-up

Participants will be followed at 3 and 6 months after the end of treatment through outpatient visits, telephone, email, home visits, or other methods. Follow-up indicators will include the VAS pain score and NDI score.

### 1.5 Observation Indicators

### 1.5.1 Observation Time Points

Assessments will be performed before treatment and after treatment, defined as after the 12th treatment session. Follow-up observations will be performed at 3 and 6 months after the end of treatment.

### 1.5.2 Observation Items

**1.5.2.1** General information: sex, age, disease course, medical history, etiology, and other information.

**1.5.2.2** Pain intensity: The Visual Analogue Scale (VAS) will be used. A ruler approximately 10 cm long will be marked with 10 graduations on one side. The two endpoints will be labeled 0 and 10. A score of 0 indicates no pain, and a score of 10 indicates severe pain. Higher scores indicate greater pain intensity. The scale will be used to evaluate neck pain.

**1.5.2.3** Neck functional symptom score: The Neck Disability Index (NDI) will be used. The scale includes 10 items: pain intensity, personal care, reading, headache, concentration, lifting, work, driving, sleeping, and recreation. Each item is scored from 0 to 5. The total NDI score ranges from 0, indicating no disability, to 50, indicating complete disability. Higher scores indicate more severe cervical dysfunction.

**1.5.2.4** Cervical range of motion: According to Rehabilitation Therapy Evaluation, a joint range-of-motion goniometer will be used to measure cervical flexion, extension, left and right rotation, and left and right lateral flexion.

**1.5.2.5** Elasticity of neck muscle tissue, including the trapezius, splenius capitis, and sternocleidomastoid muscles: Before and after treatment, shear-wave elastography (SWE) will be used to measure the neck muscles of all participants and obtain Young's modulus values (E values). The ultrasound equipment will be a Supersonic Aixplorer ultrasound diagnostic system manufactured in France, equipped with a 15L linear-array transducer operating at 4-15 MHz in musculoskeletal examination mode.

Measurement of neck muscle E values: The patient will be placed in the prone position, with both hands overlapped beneath the forehead. The shoulders will be fully abducted and externally rotated. The head and neck will be maintained in a relaxed neutral resting position to relax the neck muscles. The ultrasound probe will be positioned longitudinally, and the region of interest will be adjusted to completely cover the trapezius, sternocleidomastoid, and splenius capitis muscles. After a stable and clear two-dimensional ultrasound image is obtained, the system will be switched to shear-wave elastography mode. The tissue elasticity map will be observed, and the system will automatically calculate the Young's modulus value (E value) of the muscle tissue within the Q-BOX region. Measurements will be performed 3 times, and the average value will be used. The left side will be measured first, followed by the right side.

Note: The collected images and data will be organized and stored in a standardized manner. For statistical analysis, the mean of the left- and right-side values will be used.

### 1.6 Clinical Efficacy Evaluation

Clinical efficacy criteria were formulated with reference to the Criteria for Diagnosis and Evaluation of Clinical Disease Outcomes.

**Recovered:** After treatment, the child's pain has disappeared, neck appearance is normal, cervical movement is normal, and daily life is unaffected.

**Effective:** After treatment, the child's pain is markedly reduced, no obvious neck tilt is present, cervical movement is mildly restricted, and daily life is basically unaffected.

**Ineffective:** After treatment, the child's pain is only slightly reduced, the neck remains obviously tilted, cervical movement is markedly restricted, and daily life is affected to some extent.

Effectiveness rate (%) = (number of recovered cases + number of effective cases) / total number of cases x 100%.

### 1.7 Safety and Adherence Evaluation

**1.7.1 Safety:** Number of treatment-related adverse events.

**1.7.2 Adherence:** Number of discontinuations and dropouts among enrolled participants in the two groups.

### 1.8 Allocation Method for the Intervention Protocol

The allocation sequence will be generated using an SPSS macro program. The order in which participants attend the clinic will be matched with random numbers to complete group assignment. The group assignment corresponding to each sequence number will be placed in an opaque envelope to implement intervention allocation.

### 1.9 Data Collection, Management, and Analysis Methods

A standardized case report form will be used to collect study data. After all participants have signed the informed consent form, data will be collected by research personnel who have received standardized training. Baseline data will include sex, age, disease course, precipitating factors, previous medical history, imaging examination results, affected side, and previous treatment. Efficacy data will include pain score, Neck Disability Index score, cervical range of motion, Young's modulus values of target neck muscles measured by shear-wave elastography, clinical efficacy, safety, and adherence. Treatment-process data will include treatment dates, treatment completion, completion of resistance training, adverse events, and their management. At 3 and 6 months after treatment, follow-up will record pain score, Neck Disability Index score, symptom recurrence, and whether other related treatments were received.

Shear-wave elastography examinations will be performed by ultrasound examiners who have received standardized training. A standardized body position, measurement site, and operating procedure will be used. Each target muscle on each side will be measured 3 times, and the average value will be used as the final analysis value. All ultrasound images and original measurement data will be uniformly numbered and stored. Assessors should avoid becoming aware of participant group assignments whenever possible.

All study data will be managed by designated personnel. Paper case report forms should be completed fully, clearly, and promptly. Electronic data will be entered independently by two persons. After data entry, the data manager will check the entries. If inconsistencies are found, the original case report forms will be reviewed. Logical and range checks will be performed during data entry, including checks of age range, score range, number of treatments, follow-up time, and ultrasound measurement ranges. Participant-identifying information will be stored separately from research data. The dataset used for statistical analysis will retain only the participant identifier, group information, and study variables.

Reasons for discontinuation, dropout, and loss to follow-up will be recorded in detail during the study. All randomized participants will be included in the intention-to-treat analysis. Participants who complete the prescribed treatment course, have complete primary outcome data, and have no major protocol violations will be included in the per-protocol analysis. All participants who receive at least one treatment session will be included in the safety analysis.

### 1.10 Statistical Methods

SPSS software version 22.0 will be used for statistical analysis. All statistical tests will be two-sided. P < 0.05 will indicate statistical significance. Continuous variables will first be tested for normality. Normally distributed data will be expressed as mean +/- standard deviation. Within-group comparisons before and after treatment will use paired t tests, and between-group comparisons will use independent-samples t tests. Non-normally distributed data will be expressed as median and interquartile range. Within-group comparisons will use the Wilcoxon signed-rank test, and between-group comparisons will use the Mann-Whitney U test. Count data will be expressed as numbers and percentages, and between-group comparisons will use the chi-square test or Fisher's exact test.

The primary efficacy indicator will be the change in Young's modulus values of the target neck muscles measured by shear-wave elastography at the end of treatment. The primary analysis will compare the between-group difference in change from before to after treatment. If necessary, analysis of covariance will be used, with the post-treatment value as the dependent variable, treatment group as the fixed factor, and the baseline value as the covariate. Pain score, Neck Disability Index score, cervical range of motion, and clinical efficacy will be analyzed as secondary efficacy indicators. Clinical efficacy will be classified as recovered, effective, or ineffective. The total effectiveness rate will be calculated as the number of recovered cases plus effective cases divided by the total number of cases. Safety analysis will include the type, incidence, severity, and management of adverse events. Adherence analysis will include treatment completion rate, follow-up completion rate, discontinuation, and dropout. If primary outcome data are missing, the reasons for missingness will be described and sensitivity analysis will be performed.

# Complete English Translation of the Ethics Approval Document

Document code: Ethics Review Document A/04.01, Version 2.0

Institution: Scientific Research Ethics Committee, Hangzhou Hospital of Traditional Chinese Medicine

Document title: Ethics Review Approval

| **Project title** | A Clinical Study Using SWE to Explore Multidimensional Resistance Training Based on Joint Mobilization for the Treatment of Pediatric Atlantoaxial Subluxation |
| --- | --- |
| **Approval number** | 2024KLL088 |
| **Application status** | New project (checked); revision (not checked); amended version (not checked) |
| **Review type** | Routine review (checked); expedited review (not checked); urgent review (not checked) |
| **Investigator** | Ge Cai |
| **Category** | Scientific research |

## Review Documents

**1.** Ethics application form.

**2.** Principal investigator responsibility statement.

**3.** Research team member statement.

**4.** Clinical research protocol (Version 2.0).

**5.** Informed consent form (Version 2.0).

**6.** Application for waiver of informed consent (not applicable/not submitted).

**7.** Data management and assurance agreement (not applicable/not submitted).

## Notes

**1.** The study must comply with the ethical principles set out in applicable laws, regulations, and rules.

**2.** The clinical research protocol, informed consent form, recruitment materials, and other documents reviewed and approved by the Ethics Committee must be used in the study. Any amendment to the approved documents must be submitted to the Ethics Committee for review and approval before implementation.

**3.** If any serious adverse event (SAE) or suspected unexpected serious adverse reaction (SUSAR) occurs at Hangzhou Hospital of Traditional Chinese Medicine or at a collaborating institution, or if a safety update report is required under NMPA/GCP requirements, it must be submitted to the Ethics Committee in a timely manner.

**4.** The Ethics Committee may make a new decision on the study based on the reported circumstances.

**5.** If the investigator cannot conduct the study according to the protocol approved by the Ethics Committee, or if circumstances arise that may increase risk to participants, the investigator must promptly submit a written report to the Ethics Committee.

**6.** If a participant who does not meet the inclusion criteria or meets an exclusion criterion has already been enrolled; if a participant meets the study termination criteria but has not been withdrawn; or if a protocol deviation is implemented to eliminate an immediate hazard and protect participant rights and safety, the investigator must submit a report in accordance with GCP principles.

**7.** Any change during the conduct of the study requires submission of a protocol amendment application, and the study may proceed only after approval.

**8.** After completion of the clinical study, the applicant must submit a study completion report.

**9.** If the project involves the collection, preservation, use, or external provision of human genetic resources, approval or filing by the Human Genetic Resources Administration of China must be obtained before the study may be conducted at this center.

**10.** After project initiation, the project must be registered and filed in the Medical Research Registration and Filing Information System of the National Health Commission.

**11.** This approval document is valid for three years from the date of signature.

## Review Opinion

Approved (checked).

## Continuing Review Frequency

12 months (checked).

## Signature and Date

Chair of the Ethics Committee: Signed in the original document.

Date: 20 March 2024.

## Contact Information Shown in the Original Document

No. 453 Tiyuchang Road, Hangzhou; telephone: 0571-85827896.
