# Supplementary material for: Joint mobilization combined with cervical resistance training for pediatric atlantoaxial rotatory subluxation: Protocol for a randomized controlled trial with musculoskeletal ultrasound assessment
Source: PLoS One. 2026 Sep 24;21(9):e0359198. doi: 10.1371/journal.pone.0359198 (PMC13606960; doi:10.1371/journal.pone.0359198)
Supplement: Supplementary file 4 [file pone.0359198.s005.docx]

**Response to Reviewers**

**PLOS ONE Manuscript Revision**

**Manuscript ID:** PONE-D-26-19831

**Manuscript title:** Joint mobilization combined with cervical resistance training for pediatric atlantoaxial rotatory subluxation: protocol for a randomized controlled trial with musculoskeletal ultrasound assessment

**Corresponding author:** Haoyang Zhang

Dear Editor and Reviewer,

We sincerely thank the editor and reviewer for the careful evaluation of our manuscript and for the constructive comments. We have revised the manuscript to address all first-round journal requirements and reviewer concerns. In particular, we clarified the sample size assumptions, expanded the data management and quality-control procedures, added retention strategies and detailed missing-data procedures, specified covariate adjustment and multiplicity handling, described non-parametric and robust alternatives for model-assumption violations, and clarified how the per-protocol analysis will assess the robustness of the primary findings. We also revised the Data Availability Statement, removed Figures 3 and 4 to resolve the copyright issue, and added complete Supporting Information captions at the end of the manuscript. The financial disclosure and role-of-funders statement have been provided in the cover letter and the designated fields of the online submission system.

All changes are shown in the revised manuscript with track changes, and a clean version of the revised manuscript has also been provided.

# **Responses to Journal Requirements**

## **1. PLOS ONE style requirements and file naming**

**Comment:** *Please ensure that your manuscript meets PLOS ONE's style requirements, including those for file naming.*

**Response:** We have revised the manuscript according to the PLOS ONE formatting requirements. The main document includes a complete title page followed by the Abstract and main text. Figure citations, references, and Supporting Information captions have been checked and formatted consistently. We will upload the files using the requested labels, including “Response to Reviewers,” “Revised Manuscript with Track Changes,” and “Manuscript.”

***Location in revised manuscript:*** *Title page; Abstract; References; Supporting information*

## **2. Ethics statement only in the Methods section**

**Comment:** *Your ethics statement should only appear in the Methods section of your manuscript. If your ethics statement is written in any section besides the Methods, please delete it from any other section.*

**Response:** We have ensured that the ethics statement appears only in the Methods section under “Ethical approval and informed consent.” Any duplicated ethics-related statement outside the Methods section has been removed.

***Location in revised manuscript:*** *Methods: Ethical approval and informed consent*

## **3. Data availability statement**

**Comment:** *Please clarify how data underlying the findings will be made available after study completion and explain any restrictions on public data deposition.*

**Response:** We revised the Data Availability Statement to clarify that no datasets were generated or analyzed for the present protocol manuscript. After completion of the trial and publication of the main study results, the de-identified minimal dataset underlying the published findings, together with the data dictionary and statistical code, will be deposited in a suitable public data repository. The repository name, persistent identifier, and direct data-access information will be reported in the publication of the main trial results. Variables that cannot be safely de-identified or publicly shared because of participant confidentiality, ethical requirements, or applicable institutional regulations will be made available through a controlled-access process administered by the relevant institutional authority.

***Location in revised manuscript:*** *Data availability statement*

## **4. Role of funders**

**Comment:** *Please state what role the funders took in the study. If the funders had no role, please use the standard PLOS statement and include the amended statement in the cover letter.*

**Response:** We have provided the complete financial disclosure and the required role-of-funders statement in the cover letter and the designated fields of the online submission system. The disclosure identifies the Zhejiang Provincial Medical and Health Science and Technology Program (No. 2024KY1378), the Zhejiang Provincial Natural Science Foundation (No. LQN25H270018), and the Construction Fund of Key Medical Disciplines of Hangzhou (No. 2025HZZD18). The statement confirms that the funders had no role in study design, data collection and analysis, decision to publish, or preparation of the manuscript. Funding-related text is not included in the manuscript itself.

***Location in revised manuscript:*** *Cover letter and online submission system*

## **5. Copyright issue for Figures 3 and 4**

**Comment:** *Figures 3 and 4 contain copyrighted images. Please provide permission under the CC BY 4.0 license or remove or replace the figures.*

**Response:** We have removed Figures 3 and 4 from the revised manuscript and deleted the corresponding in-text figure citations and captions. The ultrasound assessment procedures are now described in text only. No copyrighted third-party material remains in the revised figure set.

***Location in revised manuscript:*** *Methods: Ultrasound assessment procedures; figure set*

## **6. Supporting Information captions**

**Comment:** *Please include captions for your Supporting Information files at the end of the manuscript and update any in-text citations accordingly.*

**Response:** We have added Supporting Information captions at the end of the manuscript and ensured that the in-text citations match the file labels. The revised manuscript includes “S1 File. Original ethics-approved Chinese protocol,” “S2 File. Complete English translation of the original ethics-approved clinical trial protocol,” “S3 File. SPIRIT checklist,” “S4 File. Human Participants Research Checklist,” and “S5 File. Reporting clarifications.”

***Location in revised manuscript:*** *Supporting information*

## **7. Reviewer-recommended citations**

**Comment:** *If reviewer comments include a recommendation to cite specific previously published works, please review and evaluate whether they are relevant and should be cited.*

**Response:** The reviewer did not recommend any specific previously published work for citation. We therefore focused the revision on the methodological, statistical, data-management, missing-data, and reporting issues raised during review.

# **Responses to Reviewer #1**

*General assessment: The reviewer considered the study design and analysis to be fairly well written but requested further clarification regarding the sample size assumptions, covariate adjustment, multiple comparisons, non-parametric alternatives, data management, retention strategies, missing data, and per-protocol sensitivity analysis.*

## **General comment: Sample size assumptions**

**Comment:** *The sample size is based on limited information, but may suffice for this endeavor.*

**Response:** We thank the reviewer for this observation. We agree that directly comparable pediatric AARS-specific shear-wave elastography data were limited when the trial was designed. We therefore revised the Sample size section to describe the assumptions more transparently. The calculation was informed by preliminary observations and published cervical muscle elastography studies. A target between-group difference of 6.5 kPa and a common standard deviation of 9.0 kPa were assumed, corresponding to a standardized effect size of approximately 0.72. With a two-sided significance level of 0.05, 80% power, and equal group allocation, 31 participants were required per group. Allowing for approximately 10% attrition or unavailable week-4 primary outcome data, the target sample size was set at 70 participants, with 35 participants per group. We also clarified that these assumptions should be interpreted cautiously because of the limited pediatric AARS-specific evidence and that the trial will provide estimates of treatment effects and outcome variability for future larger multicenter studies.

***Location in revised manuscript:*** *Methods: Sample size*

## **Comment 1. Covariate adjustment and multiple comparisons**

**Comment:** *Some covariate adjustment may be considered. There may be multiple comparison issues for the several secondary outcomes.*

**Response:** We revised the Statistical analysis section to clarify the planned covariate adjustment and multiplicity handling. The primary ANCOVA model will adjust for the baseline value of the primary outcome. To avoid overfitting given the modest sample size, additional covariate adjustment will be limited to prespecified clinically relevant variables. A supplementary adjusted model may include age, sex, baseline symptom duration, and baseline pain intensity if model assumptions and data completeness permit. These analyses will be considered supportive of the primary baseline-adjusted analysis. We also clarified that the primary outcome will be tested at a two-sided significance level of 0.05 without multiplicity adjustment, whereas secondary outcomes will be interpreted as supportive and exploratory. Holm-Bonferroni-adjusted P values will be reported where appropriate for secondary comparisons, together with effect estimates and 95% confidence intervals. In addition, the overall effectiveness rate at week 4 and binary recurrence-related outcomes will be compared between groups using the chi-square test or Fisher’s exact test, as appropriate.

***Location in revised manuscript:*** *Methods: Statistical analysis*

## **Comment 2. Non-parametric alternatives**

**Comment:** *Since non-parametric alternatives are mentioned for descriptive data, what non-parametric alternatives are available for the ANCOVA or linear mixed-effects models?*

**Response:** We expanded the Statistical analysis section to specify how model assumptions will be assessed and what non-parametric or robust alternatives will be considered if the assumptions of ANCOVA or linear mixed-effects models are not met. For the ANCOVA model, residual distribution, homogeneity of variance, linearity between the baseline covariate and the week-4 outcome, and influential observations will be examined using residual plots and appropriate diagnostic procedures. For linear mixed-effects models, residual distribution, variance structure, and model convergence will be assessed. If substantial deviations from model assumptions are observed, data transformation or robust standard errors will be considered where appropriate. A rank-based ANCOVA adjusted for the baseline value of the primary outcome will be used as a non-parametric sensitivity analysis if the assumptions of the standard ANCOVA are not met. For repeated secondary outcomes, transformed outcome models, robust variance estimation, or rank-based repeated-measures approaches will be considered as sensitivity analyses if linear mixed-effects model assumptions are seriously violated. The prespecified ANCOVA and linear mixed-effects models will remain the primary analytical framework.

***Location in revised manuscript:*** *Methods: Statistical analysis*

## **Comment 3. Data management and retention strategies**

**Comment:** *The Data Management section is incomplete. Since the sample size is small and this is a young cohort, a retention policy may be needed to minimize missing data.*

**Response:** We substantially expanded the data-management content and added a dedicated Retention strategies section. The revised Data collection, management and quality control section now describes standardized case report forms, password-protected electronic data entry, unique study identification numbers, separation of identifiable information from study data, independent entry of key outcome data by two trained staff members, source-document verification, range and logical-consistency checks, generation and resolution of data queries, documentation of corrections, restricted access to the final dataset, and regular data-quality checks. The Retention strategies section describes flexible appointment scheduling, telephone or electronic reminders, follow-up of missed visits within 24–48 hours, rescheduling within the prespecified assessment window, regular contact with guardians, and documentation of the reasons for missed treatment sessions, missed assessments, withdrawal, or loss to follow-up.

***Location in revised manuscript:*** *Methods: Data collection, management and quality control; Retention strategies*

## **Comment 4. Missing and inconsistent data**

**Comment:** *Please expand or explain the statement that missing or inconsistent data will be reviewed and documented. How will these issues be resolved? Will imputation be used, and how much missing data is expected?*

**Response:** We added a dedicated Missing data section. Missing data are expected to be limited, with an anticipated rate of approximately 10%, consistent with the attrition allowance used in the sample size calculation. When missing or inconsistent data are identified, the original case report forms and source documents will first be checked. Data queries will be generated and resolved by trained study personnel before database lock, and the reasons for any remaining missing data will be documented whenever possible. The intention-to-treat population will include all randomized participants, who will be analyzed according to their originally assigned groups. The primary ANCOVA will use participants with available baseline and week-4 primary outcome data. If missing primary outcome data are considered likely to influence the results, multiple imputation under the missing-at-random assumption will be performed as a sensitivity analysis. Linear mixed-effects models for repeated secondary outcomes will use all available data under the missing-at-random assumption. No imputation will be performed for adverse events, which will be summarized using observed data.

***Location in revised manuscript:*** *Methods: Missing data; Statistical analysis*

## **Comment 5. Per-protocol sensitivity analysis**

**Comment:** *The per-protocol analysis may confirm the overall intention-to-treat results, but how will it actually measure sensitivity?*

**Response:** We revised the Sensitivity analyses section to clarify how the per-protocol analysis will assess the robustness of the primary findings. The intention-to-treat analysis will be considered the primary analysis. The per-protocol population will include participants who complete the week-4 primary outcome assessment, receive at least 80% of the assigned treatment sessions, have no major protocol deviations, and do not receive prohibited co-interventions during the intervention period. Sensitivity will be evaluated by comparing the direction, magnitude, and 95% confidence intervals of the treatment effect between the intention-to-treat and per-protocol analyses. If the results are consistent across analyses, the findings will be considered robust. If meaningful discrepancies are observed, the potential influence of adherence, missing data, and protocol deviations will be examined and reported.

***Location in revised manuscript:*** *Methods: Sensitivity analyses*

# **Additional minor editorial revisions**

In addition to the changes described above, we made minor language and formatting revisions to improve clarity and consistency, including removal of repetitive text and correction of typographical and citation-formatting issues. These revisions do not alter the study design, eligibility criteria, intervention framework, outcomes, sample size, or primary analytical framework.

We again thank the editor and reviewer for their careful assessment and constructive suggestions.

Sincerely,

Haoyang Zhang
Corresponding Author
On behalf of all authors
