# Supplementary material for: Joint mobilization combined with cervical resistance training for pediatric atlantoaxial rotatory subluxation: Protocol for a randomized controlled trial with musculoskeletal ultrasound assessment
Source: PLoS One. 2026 Sep 24;21(9):e0359198. doi: 10.1371/journal.pone.0359198 (PMC13606960; doi:10.1371/journal.pone.0359198)
Supplement: Peer review history

# Peer review: Decision Letter 0

**DOI:** 10.1371/journal.pone.0359198.r001

**Authors:** Jen Edwards

8 Jul 2026

Dear Dr. Cai,

Thank you for submitting your manuscript to PLOS ONE. After careful consideration, we feel that it has merit but does not fully meet PLOS ONE’s publication criteria as it currently stands. Therefore, we invite you to submit a revised version of the manuscript that addresses the points raised during the review process.

Please note that we have only been able to secure a single reviewer to assess your manuscript. We are issuing a decision on your manuscript at this point to prevent further delays in the evaluation of your manuscript. Please be aware that the editor who handles your revised manuscript might find it necessary to invite additional reviewers to assess this work once the revised manuscript is submitted. However, we will aim to proceed on the basis of this single review if possible.

The reviewer has raised a number of concerns that need attention. They request further details on data management and a number of clarifications around multiple comparisons and missing data. Could you please revise the manuscript to carefully address the concerns raised?

Please submit your revised manuscript by Aug 22 2026 11:59PM. If you will need more time than this to complete your revisions, please reply to this message or contact the journal office at plosone@plos.org. When you're ready to submit your revision, log on to https://www.editorialmanager.com/pone/ and select the 'Submissions Needing Revision' folder to locate your manuscript file.

- A letter that responds to each point raised by the academic editor and reviewer(s). You should upload this letter as a separate file labeled 'Response to Reviewers'.
- A marked-up copy of your manuscript that highlights changes made to the original version. You should upload this as a separate file labeled 'Revised Manuscript with Track Changes'.
- An unmarked version of your revised paper without tracked changes. You should upload this as a separate file labeled 'Manuscript'.

If you would like to make changes to your financial disclosure, please include your updated statement in your cover letter. Guidelines for resubmitting your figure files are available below the reviewer comments at the end of this letter.

If applicable, we recommend that you deposit your laboratory protocols in protocols.io to enhance the reproducibility of your results. Protocols.io assigns your protocol its own identifier (DOI) so that it can be cited independently in the future. For instructions see: https://journals.plos.org/plosone/s/submission-guidelines#loc-laboratory-protocols. Additionally, PLOS ONE offers an option for publishing peer-reviewed Lab Protocol articles, which describe protocols hosted on protocols.io. Read more information on sharing protocols at https://plos.org/protocols?utm_medium=editorial-email&utm_source=authorletters&utm_campaign=protocols.

As the corresponding author, your ORCID iD is verified in the submission system and will appear in the published article. PLOS supports the use of ORCID, and we encourage all coauthors to register for an ORCID iD and use it as well. Please encourage your coauthors to verify their ORCID iD within the submission system before final acceptance, as unverified ORCID iDs will not appear in the published article. *Only*  the individual author can complete the verification step; PLOS staff *cannot*  verify ORCID iDs on behalf of authors.

We look forward to receiving your revised manuscript.

Kind regards,

Jen Edwards

Staff Editor

PLOS One

Journal Requirements:

When submitting your revision, we need you to address these additional requirements.

1. Please ensure that your manuscript meets PLOS ONE's style requirements, including those for file naming. The PLOS ONE style templates can be found at

https://journals.plos.org/plosone/s/file?id=wjVg/PLOSOne_formatting_sample_main_body.pdf and https://journals.plos.org/plosone/s/file?id=ba62/PLOSOne_formatting_sample_title_authors_affiliations.pdf

2. Your ethics statement should only appear in the Methods section of your manuscript. If your ethics statement is written in any section besides the Methods, please delete it from any other section.

3. In the online submission form, you indicated that no datasets were generated or analyzed for the present protocol manuscript. After study completion and publication of the main trial results, de-identified individual participant data that underlie the reported results may be made available on reasonable request, subject to approval by the investigators and the applicable ethical and institutional requirements. The data dictionary, statistical code, and selected supporting study materials, including the analytic output relevant to the primary report, may also be made available on reasonable request where appropriate. Requests should be directed to the corresponding author or sponsor contact and will be reviewed in relation to participant confidentiality, data-use feasibility, and local regulatory requirements.

All PLOS journals now require all data underlying the findings described in their manuscript to be freely available to other researchers, either 1. In a public repository, 2. Within the manuscript itself, or 3. Uploaded as supplementary information.

This policy applies to all data except where public deposition would breach compliance with the protocol approved by your research ethics board. If your data cannot be made publicly available for ethical or legal reasons (e.g., public availability would compromise patient privacy), please explain your reasons on resubmission and your exemption request will be escalated for approval.

4. Thank you for stating the following financial disclosure:

The authors declare that financial support was received for the research, authorship and publication of this article. This study was supported by the Zhejiang Provincial Medical and Health Science and Technology Program (No. 2024KY1378), the Zhejiang Provincial Natural Science Foundation (No. LQN25H270018), and the Construction Fund of Key Medical Disciplines of Hangzhou (No. 2025HZZD18).

Please state what role the funders took in the study. If the funders had no role, please state: "The funders had no role in study design, data collection and analysis, decision to publish, or preparation of the manuscript."

If this statement is not correct you must amend it as needed.

Please include this amended Role of Funder statement in your cover letter; we will change the online submission form on your behalf.

5. We note that Figure(s) 3, 4 in your submission contain copyrighted images. All PLOS content is published under the Creative Commons Attribution License (CC BY 4.0), which means that the manuscript, images, and Supporting Information files will be freely available online, and any third party is permitted to access, download, copy, distribute, and use these materials in any way, even commercially, with proper attribution. For more information, see our copyright guidelines: http://journals.plos.org/plosone/s/licenses-and-copyright.

We require you to either (1) present written permission from the copyright holder to publish these figures specifically under the CC BY 4.0 license, or (2) remove the figures from your submission:

a. You may seek permission from the original copyright holder of Figure(s) 3, 4 to publish the content specifically under the CC BY 4.0 license.

We recommend that you contact the original copyright holder with the Content Permission Form (http://journals.plos.org/plosone/s/file?id=7c09/content-permission-form.pdf) and the following text:

“I request permission for the open-access journal PLOS ONE to publish XXX under the Creative Commons Attribution License (CCAL) CC BY 4.0 (http://creativecommons.org/licenses/by/4.0/). Please be aware that this license allows unrestricted use and distribution, even commercially, by third parties. Please reply and provide explicit written permission to publish XXX under a CC BY license and complete the attached form.”

Please upload the completed Content Permission Form or other proof of granted permissions as an "Other" file with your submission.

In the figure caption of the copyrighted figure, please include the following text: “Reprinted from [ref] under a CC BY license, with permission from [name of publisher], original copyright [original copyright year].”

b. If you are unable to obtain permission from the original copyright holder to publish these figures under the CC BY 4.0 license or if the copyright holder’s requirements are incompatible with the CC BY 4.0 license, please either i) remove the figure or ii) supply a replacement figure that complies with the CC BY 4.0 license. Please check copyright information on all replacement figures and update the figure caption with source information. If applicable, please specify in the figure caption text when a figure is similar but not identical to the original image and is therefore for illustrative purposes only.

6. Please include captions for your Supporting Information files at the end of your manuscript, and update any in-text citations to match accordingly. Please see our Supporting Information guidelines for more information: http://journals.plos.org/plosone/s/supporting-information.

7. If the reviewer comments include a recommendation to cite specific previously published works, please review and evaluate these publications to determine whether they are relevant and should be cited. There is no requirement to cite these works unless the editor has indicated otherwise.

[Note: HTML markup is below. Please do not edit.]

Reviewers' comments:

Reviewer's Responses to Questions

**Comments to the Author**

1. Does the manuscript provide a valid rationale for the proposed study, with clearly identified and justified research questions?

Reviewer #1: Yes

**********

2. Is the protocol technically sound and planned in a manner that will lead to a meaningful outcome and allow testing the stated hypotheses?

Reviewer #1: Partly

**********

3. Is the methodology feasible and described in sufficient detail to allow the work to be replicable?

Reviewer #1: Yes

**********

4. Have the authors described where all data underlying the findings will be made available when the study is complete?

The PLOS Data policy

Reviewer #1: Yes

**********

5. Is the manuscript presented in an intelligible fashion and written in standard English?

Reviewer #1: Yes

**********

Please use the space provided to explain your answers to the questions above and, if applicable, provide comments about issues authors must address before this protocol can be accepted for publication. You may also include additional comments for the author, including concerns about research or publication ethics.

You may also provide optional suggestions and comments to authors that they might find helpful in planning their study.

Reviewer #1: Ther study design and analysis is fairly well written for this effort. However, there are some details needed as follows:

The sample size is based on limited information, but may suffice for this endeavor.

The primary outcome will be analyzed using base line adjusted ANCOVA and Linear mixed-effects models will be used for repeated secondary outcomes, with terms for group, time, and the group-by-time interaction, where appropriate.

1. Some Covariate adjustment may be considered here. There may be multiple comparison issues for the several secondary outcomes.?

2. Since some non parametric alternatives are mentioned for the descriptive data, what if any non parametric alternatives are available for the ANCOVA or Linear mixed models?

The Data Management section is incomplete.

3.Since sample size so small and this is a young cohort one may need a retention policy to minimize missing data.

4. Please expand or explain the statement: Missing or inconsistent data will be reviewed by the study team and documented. How will this be resolved? (imputation etc. ?). How much missing data is expected?

The investigators note that, Sensitivity analyses may include a per-protocol analysis. The PP analysis may confirm the overall ITT results, but

5. How will it actually measure sensitivity?

Please clarify the above issues?

**********

what does this mean?). If published, this will include your full peer review and any attached files.

If you choose “no”, your identity will remain anonymous but your review may still be made public.

**Do you want your identity to be public for this peer review?**  For information about this choice, including consent withdrawal, please see our Privacy Policy

Reviewer #1: No

**********

[NOTE: If reviewer comments were submitted as an attachment file, they will be attached to this email and accessible via the submission site. Please log into your account, locate the manuscript record, and check for the action link "View Attachments". If this link does not appear, there are no attachment files.]

To ensure your figures meet our technical requirements, please review our figure guidelines: https://journals.plos.org/plosone/s/figures

You may also use PLOS’s free figure tool, NAAS, to help you prepare publication quality figures: https://journals.plos.org/plosone/s/figures#loc-tools-for-figure-preparation.

NAAS will assess whether your figures meet our technical requirements by comparing each figure against our figure specifications.

# Author comment: Author response to Decision Letter 1

**DOI:** 10.1371/journal.pone.0359198.r002

16 Jul 2026

We sincerely thank the editor and reviewer for the careful evaluation of our manuscript and for the constructive comments. We have revised the manuscript to address all first-round journal requirements and reviewer concerns. In particular, we clarified the sample size assumptions, expanded the data management and quality-control procedures, added retention strategies and detailed missing-data procedures, specified covariate adjustment and multiplicity handling, described non-parametric and robust alternatives for model-assumption violations, and clarified how the per-protocol analysis will assess the robustness of the primary findings. We also revised the Data Availability Statement, removed Figures 3 and 4 to resolve the copyright issue, and added complete Supporting Information captions at the end of the manuscript. The financial disclosure and role-of-funders statement have been provided in the cover letter and the designated fields of the online submission system.

All changes are shown in the revised manuscript with track changes, and a clean version of the revised manuscript has also been provided.

Responses to Journal Requirements

1. PLOS ONE style requirements and file naming

Comment: Please ensure that your manuscript meets PLOS ONE's style requirements, including those for file naming.

Response: We have revised the manuscript according to the PLOS ONE formatting requirements. The main document includes a complete title page followed by the Abstract and main text. Figure citations, references, and Supporting Information captions have been checked and formatted consistently. We will upload the files using the requested labels, including “Response to Reviewers,” “Revised Manuscript with Track Changes,” and “Manuscript.”

Location in revised manuscript: Title page; Abstract; References; Supporting information

2. Ethics statement only in the Methods section

Comment: Your ethics statement should only appear in the Methods section of your manuscript. If your ethics statement is written in any section besides the Methods, please delete it from any other section.

Response: We have ensured that the ethics statement appears only in the Methods section under “Ethical approval and informed consent.” Any duplicated ethics-related statement outside the Methods section has been removed.

Location in revised manuscript: Methods: Ethical approval and informed consent

3. Data availability statement

Comment: Please clarify how data underlying the findings will be made available after study completion and explain any restrictions on public data deposition.

Response: We revised the Data Availability Statement to clarify that no datasets were generated or analyzed for the present protocol manuscript. After completion of the trial and publication of the main study results, the de-identified minimal dataset underlying the published findings, together with the data dictionary and statistical code, will be deposited in a suitable public data repository. The repository name, persistent identifier, and direct data-access information will be reported in the publication of the main trial results. Variables that cannot be safely de-identified or publicly shared because of participant confidentiality, ethical requirements, or applicable institutional regulations will be made available through a controlled-access process administered by the relevant institutional authority.

Location in revised manuscript: Data availability statement

4. Role of funders

Comment: Please state what role the funders took in the study. If the funders had no role, please use the standard PLOS statement and include the amended statement in the cover letter.

Response: We have provided the complete financial disclosure and the required role-of-funders statement in the cover letter and the designated fields of the online submission system. The disclosure identifies the Zhejiang Provincial Medical and Health Science and Technology Program (No. 2024KY1378), the Zhejiang Provincial Natural Science Foundation (No. LQN25H270018), and the Construction Fund of Key Medical Disciplines of Hangzhou (No. 2025HZZD18). The statement confirms that the funders had no role in study design, data collection and analysis, decision to publish, or preparation of the manuscript. Funding-related text is not included in the manuscript itself.

Location in revised manuscript: Cover letter and online submission system

5. Copyright issue for Figures 3 and 4

Comment: Figures 3 and 4 contain copyrighted images. Please provide permission under the CC BY 4.0 license or remove or replace the figures.

Response: We have removed Figures 3 and 4 from the revised manuscript and deleted the corresponding in-text figure citations and captions. The ultrasound assessment procedures are now described in text only. No copyrighted third-party material remains in the revised figure set.

Location in revised manuscript: Methods: Ultrasound assessment procedures; figure set

6. Supporting Information captions

Comment: Please include captions for your Supporting Information files at the end of the manuscript and update any in-text citations accordingly.

Response: We have added Supporting Information captions at the end of the manuscript and ensured that the in-text citations match the file labels. The revised manuscript includes “S1 File. Original ethics-approved Chinese protocol,” “S2 File. Complete English translation of the original ethics-approved clinical trial protocol,” “S3 File. SPIRIT checklist,” “S4 File. Human Participants Research Checklist,” and “S5 File. Reporting clarifications.”

Location in revised manuscript: Supporting information

7. Reviewer-recommended citations

Comment: If reviewer comments include a recommendation to cite specific previously published works, please review and evaluate whether they are relevant and should be cited.

Response: The reviewer did not recommend any specific previously published work for citation. We therefore focused the revision on the methodological, statistical, data-management, missing-data, and reporting issues raised during review.

Responses to Reviewer #1

General assessment: The reviewer considered the study design and analysis to be fairly well written but requested further clarification regarding the sample size assumptions, covariate adjustment, multiple comparisons, non-parametric alternatives, data management, retention strategies, missing data, and per-protocol sensitivity analysis.

General comment: Sample size assumptions

Comment: The sample size is based on limited information, but may suffice for this endeavor.

Response: We thank the reviewer for this observation. We agree that directly comparable pediatric AARS-specific shear-wave elastography data were limited when the trial was designed. We therefore revised the Sample size section to describe the assumptions more transparently. The calculation was informed by preliminary observations and published cervical muscle elastography studies. A target between-group difference of 6.5 kPa and a common standard deviation of 9.0 kPa were assumed, corresponding to a standardized effect size of approximately 0.72. With a two-sided significance level of 0.05, 80% power, and equal group allocation, 31 participants were required per group. Allowing for approximately 10% attrition or unavailable week-4 primary outcome data, the target sample size was set at 70 participants, with 35 participants per group. We also clarified that these assumptions should be interpreted cautiously because of the limited pediatric AARS-specific evidence and that the trial will provide estimates of treatment effects and outcome variability for future larger multicenter studies.

Location in revised manuscript: Methods: Sample size

Comment 1. Covariate adjustment and multiple comparisons

Comment: Some covariate adjustment may be considered. There may be multiple comparison issues for the several secondary outcomes.

Response: We revised the Statistical analysis section to clarify the planned covariate adjustment and multiplicity handling. The primary ANCOVA model will adjust for the baseline value of the primary outcome. To avoid overfitting given the modest sample size, additional covariate adjustment will be limited to prespecified clinically relevant variables. A supplementary adjusted model may include age, sex, baseline symptom duration, and baseline pain intensity if model assumptions and data completeness permit. These analyses will be considered supportive of the primary baseline-adjusted analysis. We also clarified that the primary outcome will be tested at a two-sided significance level of 0.05 without multiplicity adjustment, whereas secondary outcomes will be interpreted as supportive and exploratory. Holm-Bonferroni-adjusted P values will be reported where appropriate for secondary comparisons, together with effect estimates and 95% confidence intervals. In addition, the overall effectiveness rate at week 4 and binary recurrence-related outcomes will be compared between groups using the chi-square test or Fisher’s exact test, as appropriate.

Location in revised manuscript: Methods: Statistical analysis

Comment 2. Non-parametric alternatives

Comment: Since non-parametric alternatives are mentioned for descriptive data, what non-parametric alternatives are available for the ANCOVA or linear mixed-effects models?

Response: We expanded the Statistical analysis section to specify how model assumptions will be assessed and what non-parametric or robust alternatives will be considered if the assumptions of ANCOVA or linear mixed-effects models are not met. For the ANCOVA model, residual distribution, homogeneity of variance, linearity between the baseline covariate and the week-4 outcome, and influential observations will be examined using residual plots and appropriate diagnostic procedures. For linear mixed-effects models, residual distribution, variance structure, and model convergence will be assessed. If substantial deviations from model assumptions are observed, data transformation or robust standard errors will be considered where appropriate. A rank-based ANCOVA adjusted for the baseline value of the primary outcome will be used as a non-parametric sensitivity analysis if the assumptions of the standard ANCOVA are not met. For repeated secondary outcomes, transformed outcome models, robust variance estimation, or rank-based repeated-measures approaches will be considered as sensitivity analyses if linear mixed-effects model assumptions are seriously violated. The prespecified ANCOVA and linear mixed-effects models will remain the primary analytical framework.

Location in revised manuscript: Methods: Statistical analysis

Comment 3. Data management and retention strategies

Comment: The Data Management section is incomplete. Since the sample size is small and this is a young cohort, a retention policy may be needed to minimize missing data.

Response: We substantially expanded the data-management content and added a dedicated Retention strategies section. The revised Data collection, management and quality control section now describes standardized case report forms, password-protected electronic data entry, unique study identification numbers, separation of identifiable information from study data, independent entry of key outcome data by two trained staff members, source-document verification, range and logical-consistency checks, generation and resolution of data queries, documentation of corrections, restricted access to the final dataset, and regular data-quality checks. The Retention strategies section describes flexible appointment scheduling, telephone or electronic reminders, follow-up of missed visits within 24–48 hours, rescheduling within the prespecified assessment window, regular contact with guardians, and documentation of the reasons for missed treatment sessions, missed assessments, withdrawal, or loss to follow-up.

Location in revised manuscript: Methods: Data collection, management and quality control; Retention strategies

Comment 4. Missing and inconsistent data

Comment: Please expand or explain the statement that missing or inconsistent data will be reviewed and documented. How will these issues be resolved? Will imputation be used, and how much missing data is expected?

Response: We added a dedicated Missing data section. Missing data are expected to be limited, with an anticipated rate of approximately 10%, consistent with the attrition allowance used in the sample size calculation. When missing or inconsistent data are identified, the original case report forms and source documents will first be checked. Data queries will be generated and resolved by trained study personnel before database lock, and the reasons for any remaining missing data will be documented whenever possible. The intention-to-treat population will include all randomized participants, who will be analyzed according to their originally assigned groups. The primary ANCOVA will use participants with available baseline and week-4 primary outcome data. If missing primary outcome data are considered likely to influence the results, multiple imputation under the missing-at-random assumption will be performed as a sensitivity analysis. Linear mixed-effects models for repeated secondary outcomes will use all available data under the missing-at-random assumption. No imputation will be performed for adverse events, which will be summarized using observed data.

Location in revised manuscript: Methods: Missing data; Statistical analysis

Comment 5. Per-protocol sensitivity analysis

Comment: The per-protocol analysis may confirm the overall intention-to-treat results, but how will it actually measure sensitivity?

Response: We revised the Sensitivity analyses section to clarify how the per-protocol analysis will assess the robustness of the primary findings. The intention-to-treat analysis will be considered the primary analysis. The per-protocol population will include participants who complete the week-4 primary outcome assessment, receive at least 80% of the assigned treatment sessions, have no major protocol deviations, and do not receive prohibited co-interventions during the intervention period. Sensitivity will be evaluated by comparing the direction, magnitude, and 95% confidence intervals of the treatment effect between the intention-to-treat and per-protocol analyses. If the results are consistent across analyses, the findings will be considered robust. If meaningful discrepancies are observed, the potential influence of adherence, missing data, and protocol deviations will be examined and reported.

Location in revised manuscript: Methods: Sensitivity analyses

Additional minor editorial revisions

In addition to the changes described above, we made minor language and formatting revisions to improve clarity and consistency, including removal of repetitive text and correction of typographical and citation-formatting issues. These revisions do not alter the study design, eligibility criteria, intervention framework, outcomes, sample size, or primary analytical framework.

We again thank the editor and reviewer for their careful assessment and constructive suggestions.

Sincerely,

Ge Cai and coauthors

Attachment Submitted filename: Response to Reviewers.docx

# Peer review: Decision Letter 1

**DOI:** 10.1371/journal.pone.0359198.r003

**Authors:** Muhammad Mohsin Khan

10 Sep 2026

Joint mobilization combined with cervical resistance training for pediatric atlantoaxial rotatory subluxation: protocol for a randomized controlled trial with musculoskeletal ultrasound assessment

PONE-D-26-19831R1

Dear Dr. Ge Cai

We’re pleased to inform you that your manuscript has been judged scientifically suitable for publication and will be formally accepted for publication once it meets all outstanding technical requirements.

Within one week, you’ll receive an e-mail detailing the required amendments. When these have been addressed, you’ll receive a formal acceptance letter and your manuscript will be scheduled for publication.

An invoice will be generated when your article is formally accepted. Please note, if your institution has a publishing partnership with PLOS and your article meets the relevant criteria, all or part of your publication costs will be covered. Please make sure your user information is up-to-date by logging into Editorial Manager at Editorial Manager® and clicking the ‘Update My Information' link at the top of the page. For questions related to billing, please contact billing support.

If your institution or institutions have a press office, please notify them about your upcoming paper to help maximize its impact. If they’ll be preparing press materials, please inform our press team as soon as possible -- no later than 48 hours after receiving the formal acceptance. Your manuscript will remain under strict press embargo until 2 pm Eastern Time on the date of publication. For more information, please contact onepress@plos.org.

Kind regards,

Muhammad Mohsin Khan

Academic Editor

PLOS One

Additional Editor Comments (optional):

Reviewers' comments:

Reviewer's Responses to Questions

**Comments to the Author**

1. Does the manuscript provide a valid rationale for the proposed study, with clearly identified and justified research questions?

Reviewer #1: Yes

**********

2. Is the protocol technically sound and planned in a manner that will lead to a meaningful outcome and allow testing the stated hypotheses?

Reviewer #1: Yes

**********

3. Is the methodology feasible and described in sufficient detail to allow the work to be replicable?

Reviewer #1: Yes

**********

4. Have the authors described where all data underlying the findings will be made available when the study is complete?

The PLOS Data policy

Reviewer #1: Yes

**********

5. Is the manuscript presented in an intelligible fashion and written in standard English?

Reviewer #1: Yes

**********

Please use the space provided to explain your answers to the questions above and, if applicable, provide comments about issues authors must address before this protocol can be accepted for publication. You may also include additional comments for the author, including concerns about research or publication ethics.

You may also provide optional suggestions and comments to authors that they might find helpful in planning their study.

Reviewer #1: All comments regarding statistical concerns have been addressed and the protocol revisions noted.

XXXXXXXXXXXXXXXXXXXXXXXXXXXXXXXXXXXXXXXXXXXXXXXXXXXXXXXXXXXXXXXXXXXXXXXXXXXXXXXXXXXXXXXXXXXXXXXXXXXXXXXXXXXXXXXXXXXXXXXXXXXXXXXXXXXXXXXXXXXXXXXXXXXXXXXXXXXXxx

**********

what does this mean?). If published, this will include your full peer review and any attached files.

If you choose “no”, your identity will remain anonymous but your review may still be made public.

**Do you want your identity to be public for this peer review?**  For information about this choice, including consent withdrawal, please see our Privacy Policy

Reviewer #1: No

**********

# Editor report: Acceptance letter

**DOI:** 10.1371/journal.pone.0359198.r004

**Authors:** Muhammad Mohsin Khan

PONE-D-26-19831R1

PLOS One

Dear Dr. Cai,

I'm pleased to inform you that your manuscript has been deemed suitable for publication in PLOS One. Congratulations! Your manuscript is now being handed over to our production team.

At this stage, our production department will prepare your paper for publication. This includes ensuring the following:

* All references, tables, and figures are properly cited

* All relevant supporting information is included in the manuscript submission,

* There are no issues that prevent the paper from being properly typeset

You will receive further instructions from the production team, including instructions on how to review your proof when it is ready. Please keep in mind that we are working through a large volume of accepted articles, so please give us a few days to review your paper and let you know the next and final steps.

Lastly, if your institution or institutions have a press office, please let them know about your upcoming paper now to help maximize its impact. If they'll be preparing press materials, please inform our press team within the next 48 hours. Your manuscript will remain under strict press embargo until 2 pm Eastern Time on the date of publication. For more information, please contact onepress@plos.org.

You will receive an invoice from PLOS for your publication fee after your manuscript has reached the completed accept phase. If you receive an email requesting payment before acceptance or for any other service, this may be a phishing scheme. Learn how to identify phishing emails and protect your accounts at https://explore.plos.org/phishing.

If we can help with anything else, please email us at customercare@plos.org.

Thank you for submitting your work to PLOS One and supporting open access.

Kind regards,

PLOS One Editorial Office Staff

on behalf of

Dr. Muhammad Mohsin Khan

Academic Editor

PLOS One
